# Electro‐Sorting Create Heterogeneity: Constructing A Multifunctional Janus Film with Integrated Compositional and Microstructural Gradients for Guided Bone Regeneration

**DOI:** 10.1002/advs.202307606

**Published:** 2024-01-15

**Authors:** Miao Lei, Haitao Liao, Shijia Wang, Hang Zhou, Jianwei Zhu, Haoran Wan, Gregory F. Payne, Changsheng Liu, Xue Qu

**Affiliations:** ^1^ Key Laboratory for Ultrafine Materials of Ministry of Education Frontiers Science Center for Materiobiology and Dynamic Chemistry School of materials science and engineering East China University of Science and Technology Shanghai 200237 China; ^2^ Institute for Bioscience and Biotechnology Research and Robert E. Fischell Biomedical Device Institute 5118 A. James Clark Hall, College Park Maryland 20742 USA; ^3^ Shanghai Frontier Science Research Base of Optogenetic Techniques for Cell Metabolism East China University of Science and Technology Shanghai 200237 China

**Keywords:** controlled‐heterogeneity, electro‐sorting, gradient composition, guided bone regeneration, janus porous structure

## Abstract

Biology remains the envy of flexible soft matter fabrication because it can satisfy multiple functional needs by organizing a small set of proteins and polysaccharides into hierarchical systems with controlled heterogeneity in composition and microstructure. Here, it is reported that controlled, mild electronic inputs (<10 V; <20 min) induce a homogeneous gelatin‐chitosan mixture to undergo sorting and bottom‐up self‐assembly into a Janus film with compositional gradient (i.e., from chitosan‐enriched layer to chitosan/gelatin‐contained layer) and tunable dense‐porous gradient microstructures (e.g., porosity, pore size, and ratio of dense to porous layers). This Janus film performs is shown multiple functions for guided bone regeneration: the integration of compositional and microstructural features confers flexible mechanics, asymmetric properties for interfacial wettability, molecular transport (directional growth factor release), and cellular responses (prevents fibroblast infiltration but promotes osteoblast growth and differentiation). Overall, this work demonstrates the versatility of electrofabrication for the customized manufacturing of functional gradient soft matter.

## Graphical abstract

Electronic inputs can “sort” a homogeneous mixture of bio‐polymers (i.e., gelatin and chitosan) and induce self‐assembly to generate flexible Janus films with gradients in composition and microstructure. Films with tailored composition and microstructure are shown to perform the multiple functions needed to guide bone regeneration. Overall, this work demonstrates the versatility of electrofabrication in manufacturing soft materials with controlled heterogeneity.

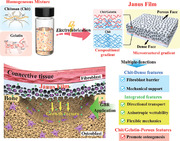

## 1 Introduction

Controlled heterogeneity (e.g., gradients) is a prevalent feature in biological systems, enabling diverse morphologies and functions to be generated from a small set of proteins and polysaccharides. One class of controlled heterogeneity originates from the local variations in the content of molecular types or concentration (i.e., compositional gradients). Examples include the arthropods cuticle, where the mechanically strong, chitin‐rich outer surface for protection transitions to a compliant, protein‐rich inner surface that connects to the body;^[^ ^1^ ^]^ Beetle setae, where the hard, chitin‐rich bases for preventing their lateral collapse transition to the soft, elastin‐rich tips for enlarging the contact area to aid adhesion.^[^ ^2^ ^]^ Another class of controlled heterogeneity involves spatial changes in morphological structure (i.e., microstructural gradients), such as pore size and porosity. For instance, bone transitions from a dense, non‐porous exterior (i.e., cortical bone) to a loose and porous interior (i.e., spongy bone), and this microstructural gradient balance the need for lightweight strength while providing space for cells and tissues (e.g., blood vessels, nerves).^[^ ^3^ ^]^ Also, the stems of plants (e.g., bamboo, spruce) exhibit a microstructural gradient with a dense outer surface to confer mechanical stability and a porous interior with radially changing porosity to facilitate the directional transport of mass (e.g., water).^[^ ^4^ ^]^

While it is well‐established that the creation of materials system with compositional and microstructural gradients enables biology to flexibly customize local properties,^[^ ^5^ ^]^ recapitulating these capabilities in manufacturing is challenging.^[^ ^6^ ^]^ This is because such sophisticated gradients in biological structures typically rely on the bottom‐up, heterogeneous assembly process of biomacromolecules.^[^ ^7^ ^]^ In general, spontaneous entropy‐driven self‐assembly processes tend to form homogeneously distributed multiple‐component material systems.^[^ ^8^ ^]^ We suggest that it may be possible to create gradient materials by: (i) using biomacromolecules that possess internal structural information for bottom‐up self‐assembly; and (ii) imposing precise and exogenous cues to guide the assembly in a non‐homogeneous way.

Attempts have been made to use additive methods (e.g., 3D printing, electrospinning, controlled polymerization and freeze‐drying, etc.) and subtractive methods (e.g., gradient pore etching, etc.) to construct biomacromolecular materials with a structural (e.g., porous) gradient, while possibly controlling the compositional gradient by continuously changing the formulation of the molding substrate (e.g., printing ink).^[^ ^9^ ^]^ Typically however, these methods have limited abilities to create gradients within micrometer‐scales both in composition and microstructures, thus can only partially recapitulate the complex functional properties achieved by biology.^[^ ^6^, ^10^ ^]^

**Scheme 1:**
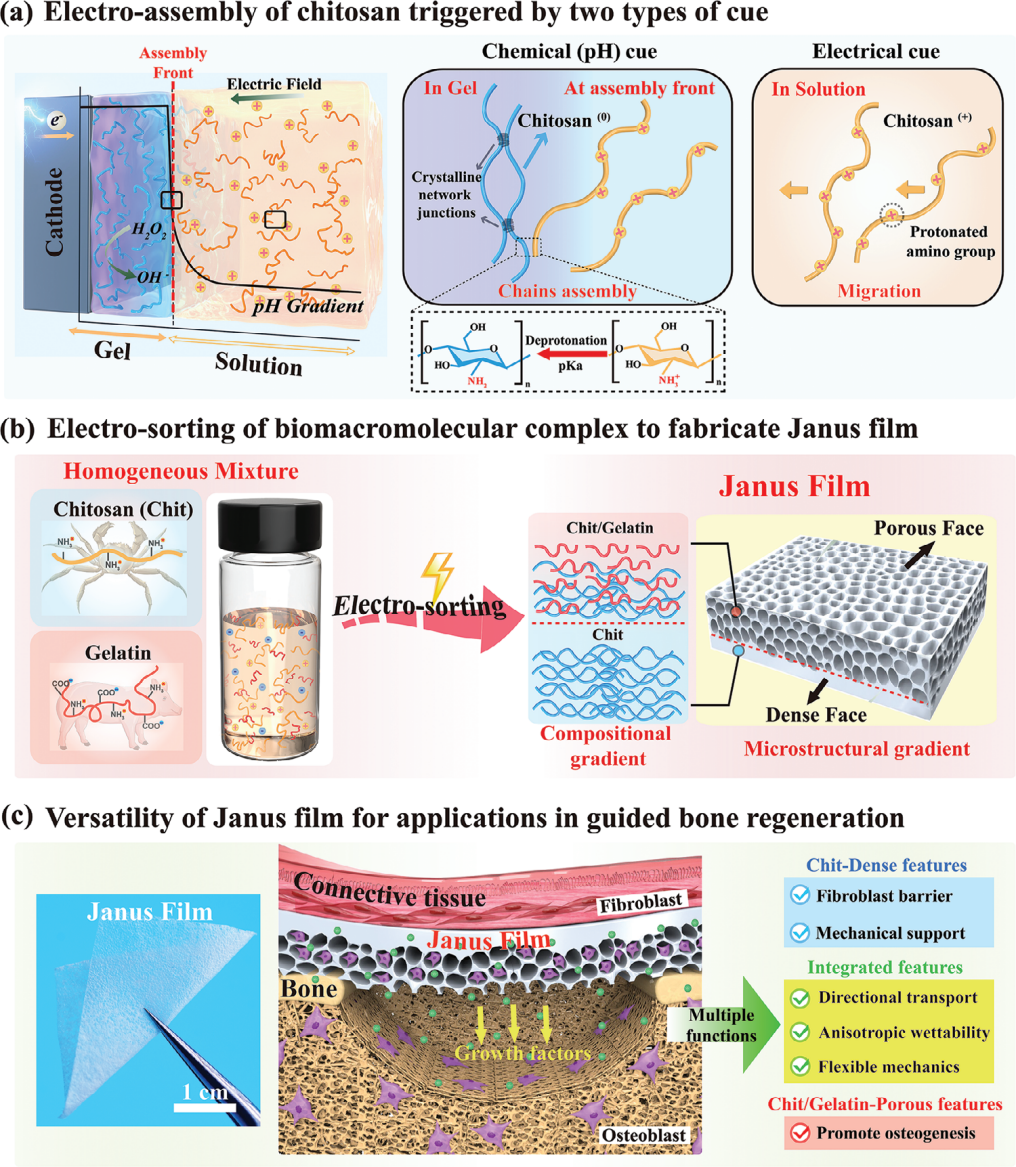
a) Electrode imposed inputs provide two cues that control the electro‐assembly of chitosan: the pH cue that arises from electrolytic reactions induces chitosan chains to deprotonate and self‐assemble; and the electric field cue induces cationic chitosan chains to migrate. b) Electronic inputs can “sort” a homogeneous mixture of the protein gelatin and the polysaccharide chitosan, and cue the emergence of a Janus film with integrated gradients in composition and microstructure. c) The Janus film with gradient features acts at the interface between connective tissue and bone, allowing it to perform various functions in guided bone regeneration: the chitosan‐enriched dense face provides mechanical support and a barrier for fibroblasts ingrowth, while the chitosan/gelatin‐porous face promotes osteoblast growth and differentiation. The gradients in composition and microstructure further endow the Janus film with directional mass transport, anisotropic wettability and flexible mechanics.

Electrofabrication is an emerging additive manufacturing approach that enlists imposed electrical signals (generally < 5 V) to guide the assembly of materials at (or near) electrode surfaces.^[^ ^11^ ^]^ Especially, electrode‐imposed signals can provide spatiotemporally‐controlled cues to assemble biomacromolecular materials with compositional and microstructural control. First, the electric field provides a cue that induces charged biomacromolecular chains to migrate toward the designated electrode, and thus selectively enriches them from solution.^[^ ^12^ ^]^ Second, electrode reactions can generate molecular cues that can induce the sol‐gel transition and generate hierarchical assembly structure (e.g., the cathodic electrolysis of H_2_O_2_ can locally generate OH^−^ ions).^[^ ^13^ ^]^ For instance, amino‐polysaccharide chitosan as a bio‐derived polycation with internal structural information for bottom‐up assembly can undergo a reversible sol‐gel phase transition by increasing the pH above its pKa. Imposing electrical signals can induce migration of charged chitosan chains to cathode, and cathodic reactions can yield high pH conditions near the electrode surface that can induce chitosan's deprotonation, and self‐assembly through the formation of physical crosslinks (e.g., crystalline network junctions), as shown in **Scheme** **1**a. Previous work demonstrates this process enables the coupling of electrical cues (e.g., current density, oscillated inputs) and electrolyte environment (e.g., ionic, temperature) to finely tune assembly of microstructure by regulating a subtle balance of macromolecular interactions (e.g., among electrostatic, hydrogen bonding and hydrophobic interactions), presenting exciting possibilities for creating microstructural gradients.^[^ ^14^ ^]^

Here we report that electronic inputs can “sort” a homogeneous dual‐composition mixture of gelatin and chitosan to enable the emergence of a Janus film with integrated gradients in composition and microstructure. Compositionally, the Janus films present a transition from a layer rich in chitosan to a layer containing both chitosan and gelatin. Structurally, this Janus film presents a dramatic gradient transitioning from a dense, non‐porous layer (i.e., enriched in chitosan) to a loose and porous layer (i.e., containing both chitosan and gelatin), as illustrated in Scheme 1b. Mechanistically, we hypothesize that chitosan and gelatin possess distinct internal structural information, leading to their differential charge characteristics under varying pH conditions. This divergence allows them to create compositional gradients through selective spatiotemporal responses to electric fields. Consequently, these resultant compositional gradients further catalyze the development of microstructural gradients by modulating local electrochemical assembly of chitosan.

Moreover, we demonstrated the versatility of this Janus film integrated compositional and microstructural gradients in a specific biomedical application (i.e., guided bone regeneration, GBR) which imposes substantial demands both in terms of the molecular type and their hierarchical structure.^[^ ^15^ ^]^ Generally, GBR materials should be selected that are intrinsically biodegradable (i.e., to avoid the need for a second surgery for removal), biocompatible (i.e., to limit inflammation) and biologically active (i.e., to promote healing).^[^ ^16^ ^]^ While synthetic polyesters (e.g., Resolut LT, Vicryl) can satisfy the biodegradability requirement, the cellular responses are often inferior while the acidic degradation‐products may trigger inflammation. Alternative, collagen‐based natural materials are also used (e.g., Bio‐Gide, AlloDerm), and while they exhibit excellent tissue compatibility, their rapid degradation leads to a premature loss of structural integrity.^[^ ^17^ ^]^ GBR also imposes significant demands on the material's microstructural organization because the material acts at the interface between connective tissue and bone, and ideally, this material should have anisotropic structural features to meet the differing functional requirements for bone regeneration. Such Janus films act between connective tissue and bone present anisotropic physicochemical local properties to endow multiple biological functions for enhancing bone regeneration: the chitosan‐rich dense non‐porous surface of the film facing connective tissue provide sufficient mechanical support to prevent film collapse and act as a barrier against fibroblast invasion of bone defects; the porous surface containing both chitosan and gelatin in the film facing the bone defect presents more biological functions, and the local coupling of composition (i.e., enriched gelatin) and microstructure (i.e., porous structure) provides a suitable microenvironment for osteoblast adhesion, proliferation and differentiation. Besides, the integrated features of gradients in composition and microstructure of Janus film endows flexible mechanics, anisotropic wettability and allows bioactive molecules directional transport to further promote bone regeneration, as shown in Scheme 1c. Here, we specifically report that imposed electrical signals can be used to sort multi‐component biomacromolecules to create materials with composition and structural gradients. We anticipate that the electro‐sorting of biomacromolecules will provides a new approach to fabricate gradient materials tailored to meet diverse applications‐specific needs.

## 2 Result and Discussion

### 2.1 Electro‐Molecular Sorting to Generate a Gradient in Composition

**Figure 1:**
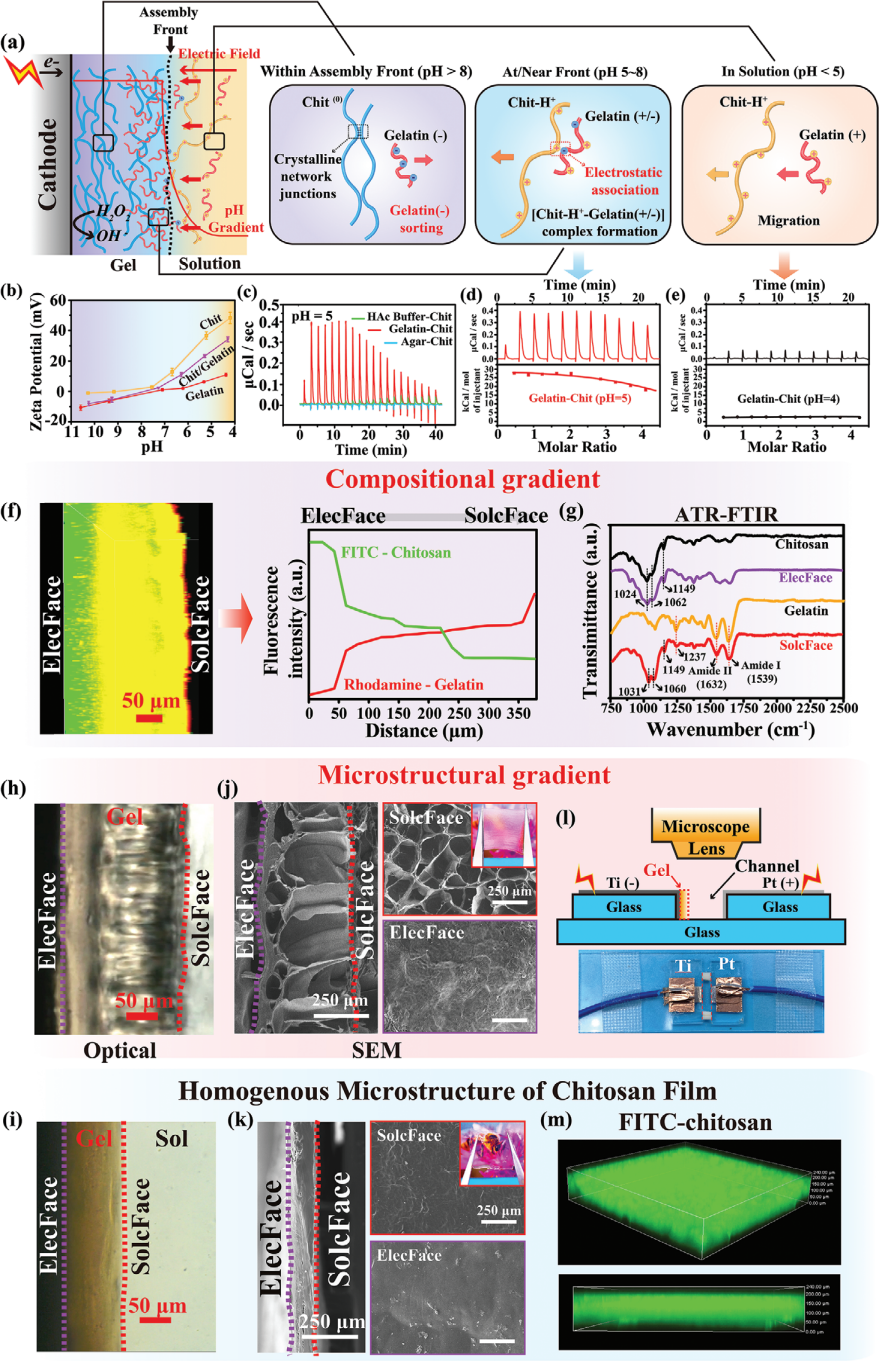
Electro‐molecular sorting from a homogeneous mixture of chitosan and gelatin to generate a film with gradients both in composition and microstructure. a) Illustration of three spatial regimes during deposition. b) pH‐dependence of the Zeta potential for chitosan and gelatin (both in 0.1% w/v) and their mixture in equal proportions. c) ITC measurements reveal an endothermic interaction between chitosan and gelatin in the intermediate pH regime (pH approx. 5; agar is used as a non‐interacting control), and d–e) the comparison of endothermic interactions between chitosan and gelatin at intermediate pH (pH ≈5) and slightly lower pH (pH ≈ 4) reveals the interaction weakens with decreasing pH. f) The high‐resolution cross‐sectional fluorescence image and corresponding image intensity shows film's compositional gradient. g) ATR‐FTIR spectra show compositional difference between the two faces of the film. In situ observation of the kinetic assembly process of h) mixture of chitosan/gelatin and i) chitosan within a fluid channel (see Movies S1 and S2 in the Supporting Information). SEM images of two surfaces and cross‐sections of the resultant j) gelatin‐chitosan film and k) chitosan film and their optical appearance. l) The schematic of the fluid channel device for the in situ observation. Reprinted (adapted) with permission from ref. [21] Copyright 2022 American Chemical Society. m) Chitosan component labeled with FITC (in green) uniformly distribute in the chitosan film.

We propose that a homogeneous solution of gelatin and chitosan can be “sorted” during cathodic electro‐assembly to induce a compositional gradient in the electro‐assembled film first. This proposed electrochemical sorting mechanism is illustrated in **Figure** **1**a. The electro‐assembly of gelatin and chitosan is initially performed at pH 4.5. Far from the electrode, this low pH solution (<5) is expected to form a homogeneous mixture of isolated polycationic chains, and both the positively charged chitosan and gelatin chains should migrate toward the cathode in response to the imposed electric field. In an intermediate pH regime (5‐8) at/near the assembly front, chitosan should retain some positive charge while some of gelatin's acidic residues (e.g., glutamate residues) should be deprotonated and become anionic. In this intermediate pH regime, chitosan and gelatin chains could form electrostatic associations and the complex would retain a net positive charge (consistent with this explanation, the photographs in Figure S1a (Supporting Information) show some aggregates appear in the gelatin/chitosan solution at pH 5.5). Thus, in locations of intermediate pH, the electric field should drive the cationic gelatin‐chitosan complex toward the interface. In the high pH regime (>8), near/within the assembled gel, chitosan should be uncharged and self‐assemble with other chitosan chains, while gelatin should be anionic and dis‐associated from chitosan or other gelatin chains. In these high pH locations, the electric field should not drive the neutral chitosan chains to move, but should drive the anionic gelatin chains to migrate away from the electrode. While Figure 1a and the above text suggests a potential mechanism to sort a homogeneous mixture of gelatin and chitosan to electro‐assemble into a film with a gradient in composition, it is important emphasize that electro‐assembly is a dynamic process involving many phenomena which occur at characteristic times that are not fully understood.

A key assumption of this proposed sorting mechanism is that the chitosan chains will transition from a protonated to a neutral state as they approach the cathode, while the gelatin chains will undergo a charge reversal transitioning from a cationic state far from the electrode to an anionic state very close to the electrode. To provide evidence for this assumption, we performed Zeta potential measurements with chitosan solutions (0.1% w/v) and gelatin solutions (0.1% w/v) and a mixture of gelatin and chitosan (0.05% w/v each) that were adjusted to different pHs. Figure 1b shows the pH‐dependence of the Zeta potential for these solutions (note: we inverted the pH scale to facilitate comparison with the electro‐assembly schematic in Figure 1a). As expected, the Zeta potential for the chitosan solution is a strong function of pH: high positive values are observed at the lowest pH and it decreases asymptotically approaching zero at the highest pHs. Also as expected, Figure 1b shows the Zeta potential for the gelatin solution is a weaker function of pH and a reversal in charge is observed.

A second assumption of the proposed sorting mechanism is that in the intermediate pH regime, the gelatin and chitosan chains will undergo electrostatic interactions to form a polyelectrolyte complex.^[^ ^18^ ^]^ To provide evidence for such gelatin‐chitosan complexation we performed isothermal titration calorimetry (ITC). For these studies we prepared individual 0.1% w/v biomacromolecular solutions of chitosan, and gelatin and agar dissolved in an acetic acid (HAc) buffer solution (1 × 10^−3^ M; pH ≈ 5) and performed measurements by titrating aliquots into the chitosan solution. Figure 1c shows large endothermic peaks were observed when the gelatin solution was titrated into the chitosan solution while no such peaks were observed for the titration of control solutions lacking gelatin or containing agar. Using estimated molecular weights for chitosan (300000) and gelatin (75000), it was possible convert these measurements into a binding affinity [(9.7 ± 2.3) ×10^5^ M^−1^] and binding enthalpy [31.5 ± 2.1 kcal mol^−1^] as shown in Figure 1d. Our observed endothermic binding enthalpy is consistent with various experimental results that show polyelectrolyte complexation is driven by entropy.^[^ ^19^ ^]^ Besides, the interaction between gelatin and chitosan is significantly weaken at a relatively low pH (≈4) compared to the pH 5 condition, as shown in Figure 1e. This result indicates that the interaction between gelatin and chitosan is pH‐dependent, and further proves another assumption in Figure 1a that chitosan and gelatin should be separated polycationic chains at low pH < 5 and without strong interaction.

To provide direct evidence for the proposed sorting mechanism, we performed electro‐assembly studies from a mixture of dye‐labeled gelatin and chitosan. Specifically, chitosan was labeled with fluorescein isothiocyanate (FITC), and gelatin was labeled with rhodamine B, and a mixture was prepared containing 0.5% of each biomacromolecule (pH 4.5; note: this electrolyte was not buffered and 0.1 M H_2_O_2_ was added to enable cathodic electrolysis to generate the pH gradient without the generation of gas bubbles). Confocal fluorescence images of this solution (Figure S1b in Supporting Information) indicate that the two biomacromolecules are homogeneously distributed throughout the solution (i.e., the interactions between gelatin and chitosan chains is weak at this low pH do not result in observable phase separation). For electro‐assembly, we used a Ti foil (2 cm × 2 cm) as our working electrode (i.e., cathode) and immersed it into the biomacromolecular mixture and applied cathodic potential (6.67 mA cm^−2^ for 1000 s; <9 V). After assembly, the electrode coated with the hydrogel film was removed from the solution, rinsed with deionized water, and peeled off the titanium foil.

This electro‐assembled film was imaged by confocal fluorescence microscopy and the high‐resolution cross‐sectional analysis of the merged image in Figure 1f shows significant gradients in the green and red fluorescence. The 50 µm region of the film that had been in direct contact with the electrode (ElecFace) shows strong green fluorescence with no significant red fluorescence which consistent with the chitosan enrichment and gelatin‐depletion from the region closest to the electrode. The intermediate, yellow region in Figure 1f indicates that both gelatin and chitosan are present in the central region of the assembled hydrogel. The outer region of the film that had been in direct contact with the solution (SolcFace) shows a thin region of red fluorescence which suggests a thin layer of gelatin is assembled at the gelation front. This analysis is consistent with the proposed sorting mechanism and indicates that electro‐assembly from a gelatin‐chitosan solution can generate an electro‐assembled film with a gradient in composition. The low magnification fluorescence images of the whole electro‐assembled film were shown in Figure S1c of Supporting Information.

To provide additional evidence for a compositional gradient between the two faces of the film, we electro‐assembled a film from a solution of gelatin and chitosan using equivalent conditions described above and analyzed the two faces using attenuated total reflectance FTIR (ATR‐FTIR). For comparison, we prepared two control films by pouring either a chitosan solution or gelatin solution into Petri dishes, dried the films at room temperature and neutralized the chitosan film by soaking in PBS (0.5 M, pH = 7.4). The ATR‐FTIR spectrum in Figure 1g for the ElecFace shows absorption bands characteristic of chitosan (e.g., C‐O stretching vibration of 1024 cm^−1^, C‐N stretching vibrations of 1062 cm^−1^ and the asymmetric C‐O‐C stretching vibrations of 1149 cm^−1^) and little absorption in regions characteristic of gelatin (the amide I and II bands at 1632 cm^−1^ and 1539 cm^−1^). In contrast, the characteristic absorption peaks of chitosan and gelatin are both observed on the SolcFace surface. These results provide further evidence that gelatin and chitosan can be sorted during electro‐assembly to generate an electro‐assembled film with a gradient in composition. It is relatively straightforward to understand how the electrode‐imposed cues can generate a gelatin/chitosan film with a gradient in composition: the pH cue induces chitosan to self‐assemble, while the electric field cue provides the selective force to create the gelatin/chitosan compositional gradient.

### 2.2 Electro‐Molecular Sorting Induce The Emergence of Janus Porous Structure

We further provide direct evidence that applied electrode cues also induce the emergence of microstructural gradient. Specifically, electro‐assembly can be performed on a sidewall electrode of a fluidic device and this allows the in situ direct observation of the hierarchical assembly and the emergent microstructure.^[^ ^20^ ^]^ Our fluidic device uses a titanium foil as the cathode and an opposing platinum foil as the anode (as shown later in Figure 1l),^[^ ^21^ ^]^ while video imaging allows a recording of the dynamics of the hierarchical assembly and the detection of microstructural features. Experimentally, the fluidic channel was filled with a mixture chitosan and gelatin solution (both in 0.5% w /v; pH 4.5; 0.1 M H_2_O_2_), the power was set to a constant current density (i.e., 6.67 mA cm^−2^) and the growth of the assembling film was observed with an optical microscope. The video (Movie S1, Supporting Information) shows that during electro‐assembly from this chitosan/gelatin mixture, a Janus microstructure (i.e., a special class of gradient structure) emerges. The optical micrograph image in Figure 1h shows a representative cross‐sectional image of an electro‐assembled chitosan/gelatin film, and the assembled film was observed to have a heterogeneous optical appearance along thickness, and shows a dramatic transition within the film at the 50 µm position that had been in direct contact with the electrode near (i.e., ElecFace), potentially suggesting the emergence of the changes in the microstructure of the assembled film along the thickness. For comparison, when we performed electro‐assembly from a single chitosan component solution under analogous conditions, the video (Movie S2, Supporting Information) and representative micrograph image show a uniform structure formation as shown in Figure 1i. To further assess the microstructure of electro‐assembled films, we prepared films by electro‐assembly from a chitosan/gelatin mixture onto Ti plates. Briefly, the Ti foil (2 cm × 2 cm) working electrode was immersed in a chitosan/gelatin solution (both in 0.5% w/v; pH 4.5; 0.1 M H_2_O_2_), and a cathode potential (6.67 mA cm^−2^, 1000 s) was applied. After assembly, the film‐coated electrode was removed from the solution, rinsed with deionized water, and the film was then peeled‐off from the titanium foil and freeze‐dried, for comparison, a chitosan hydrogel film was electro‐assembled using the same procedure. The insert in the upper right corner of Figure 1j shows this electro‐assembled gelatin/chitosan film is translucent in appearance, and the cross‐sectional SEM images on the left of Figure 1j intuitively show the internal microstructure of the electro‐assembled gelatin/chitosan film after freeze‐drying. It can be seen that the region about 50 µm of the film that had been in direct contact with the electrode (ElecFace) shows a dense structure (note: this region is also that where the chitosan is enriched in the film), while along the thickness of the film, the closer to the region of the film that contact with the solution (SolcFace), the more porous the microstructure appears (note: this region within the film contains both chitosan and gelatin). The upper and lower two SEM images on the right of Figure 1j respectively show the microstructure for the two faces of chitosan/gelatin film: the SolcFace has a honeycomb porous structure, while the ElecFace has a uniform and dense structure. For comparison, a control film assembled from a single chitosan component solution using the same procedure, and this control film is transparent and with a homogenous dense structure and distribution of component as shown in Figure 1k,m.

It is relatively difficult to understand how the imposed electrode cues can generate a gradient in microstructure. In our previous studies, we electro‐assembled a Janus chitosan film by sequentially manipulating the electric field cue. Specifically, we performed an initial assembly step to generate a dense face by electro‐assembling from a chitosan solution with minimal salt: this condition strengthens electrostatic forces and enhances chain migration to the assembly front. The porous face was then grown onto the dense face by transferring the electrode to a high salt chitosan solution and performing electro‐assembly: this condition screens electrostatic forces and limits the accumulation of chains at the assembly front.^[^ ^22^ ^]^ In the second study, we exploited a surprising experimental observation that a Janus film could spontaneously emerge in a single step if electro‐assembly was performed under sub‐ambient temperatures (5 °C). While a lower temperature is expected to enhance hydrogen bonding and suppress hydrophobic interactions, the complex dynamics observed during sub‐ambient electro‐assembly cannot be fully explained.^[^ ^23^ ^]^ Together, these studies illustrate that the emergent microstructure involves a subtle interplay between top‐down contextual cues (chemical and electrical cues) and the inter‐molecular interactions responsible for bottom‐up self‐assembly. In this work, we believe the emerged microstructure of Janus film mainly relies on chitosan's deprotonation and self‐assembly through the formation of physical crosslinks (e.g., crystalline network junctions), as earlier mentioned in Scheme 1a. The difference is that electro‐molecular sorting mechanism triggered the spatially selective distribution of gelatin in the electro‐assemble chit/gelatin two‐component film, which potentially suppress the crystalline networks formation of chitosan in the region of gelatin presence within the film (as shown in the XRD results of Figure S2a in Supporting Information), and led to the loose organization of biomacromolecular chains and induce the generation of local porous structure. [Note: the decreased storage modulus (G’) and loss modulus (G’’) of chit/gelatin Janus film, as shown in the rheology results of Figure S2b in Supporting Information]

### 2.3 Gelatin Tuning of The Generated Porous Structure of Janus Film

**Figure 2:**
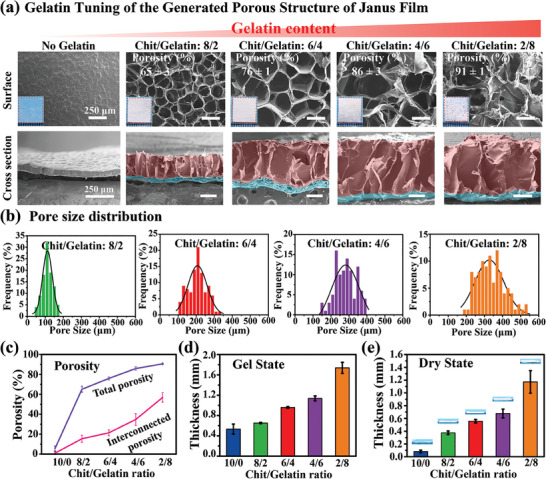
Tuning the porous structure of the Janus Chit/Gelatin film by changing gelatin content. a) Optical (inset in upper row) and SEM images of Janus Chit/Gelatin films (the dense layer is marked by pseudo‐blue color, and porous layer is marked by pseudo‐red color) show how the gelatin content of the electrolyte solution affects important structural features of the porous layer. b) Histograms of pore size distribution for the SolcFace surface of Janus Chit/Gelatin films (n = 100) with different mass ratios of Chit/Gelatin. c) The total and interconnected porosity of films with varying mass ratios of Chit/Gelatin. The thickness of films with varying mass ratios of Chit/Gelatin in the d) gel state and e) dry state (all films deposited under the same conditions of 6.67 mA cm^−2^ and 1000 sec). (n = 6). (Note: none of the samples characterized above have been chemically crosslinked).

Presumably, the Janus porous structure emerged for the chitosan/gelatin film due to subtle sorting of gelatin through the electric field when chitosan was electro‐assembled in the presence of gelatin. To further demonstrate the correlation between the inclusion of gelatin and the formation of porous structure, we changed the gelatin content of the electro‐assembly solution in a subsequent studies, and the results showed that this manner systematically changed the porous microstructure of the film. This systematic variation is illustrated by experiments in which electro‐assembly was performed from 1% w/v biomacromolecular solutions (pH 4.5) containing different mass ratios of chitosan and gelatin. Specifically, electro‐assembly was performed using titanium foil electrodes (2 cm × 2 cm) at a constant current density of 6.67 mA cm^−2^ for 1000 s. The optical images in **Figure** **2**a (inset) show the film prepared from chitosan (Chit/Gelatin is 10/0) is transparent, while films electro‐assembly from gelatin‐containing solutions are opaque, which may be due to the different scattering of light that related to the films’ internal microstructures.

After freeze‐drying, the films were observed by SEM (Figure 2a). The SEM images of the surfaces of the SolcFace show the inclusion of gelatin in the electro‐assembly solution resulted in the formation of a porous microstructure with important features systematically varying with the gelatin content (e.g., the pore size and porosity). The pore size distribution of the SolcFace was analyzed using Image J Plus software and Figure 2b shows that as the chit/gelatin ratio was increased, the pore size of the SolcFace systematically increased from ≈100 µm to 300 µm. In addition, we measured the total porosity and the interconnected porosity of all films using a gravimetric method and an ethanol‐wicking technique, respectively. Figure 2c shows that as the chit/gelatin ratio increased, the total porosity of the film increased from ≈65% to 91%, and the interconnected porosity showed the similar increasing trend. The inclusion of gelatin into the electro‐assembly solution not only altered the pore structure of the porous layer, but also the relative thicknesses of the dense and porous layers. The cross‐sectional SEM images in Figure 2a highlight the porous layer with a pseudo‐red color and the dense layer with a pseudo‐blue color. These images illustrate that as the gelatin content of the electro‐assembly solution was increased, the porous layer became thicker while the thickness of the dense layer decreases slightly.

Finally, the thickness of the electro‐assembled hydrogel film, both before and after drying, was quantified using an optical microscope and SEM, respectively. Figure 2d,e show that thicker films were generated when gelatin was incorporated into the electro‐assembly solution despite the fact that all these gels were assembled using the same electrical input (6.67 mA cm^−2^ for 1000 s; 6.67 C cm^−2^). Previous studies have shown that when electrostatic forces are suppressed (e.g., by adding salt to the electro‐assembly solution) chitosan's slower migration under the same electrical inputs leads to the rapid growth of thicker, more porous gels.^[^ ^22^, ^24^ ^]^ Possibly, the inclusion of gelatin in the electro‐assembly solution and the formation of chitosan‐gelatin complexes suppress the electrostatic forces beyond the 50 µm region where the dense chitosan‐rich film is assembled.

In summary, the results in Figure 2 demonstrate that electro‐assembly from a chitosan/gelatin homogenous mixture leads to the formation of a Janus porous film with important features of the gradient microstructure controlled by the ratio of chitosan to gelatin.

### 2.4 Flexible Mechanics and Anisotropic Wettability of Janus Film

**Figure 3:**
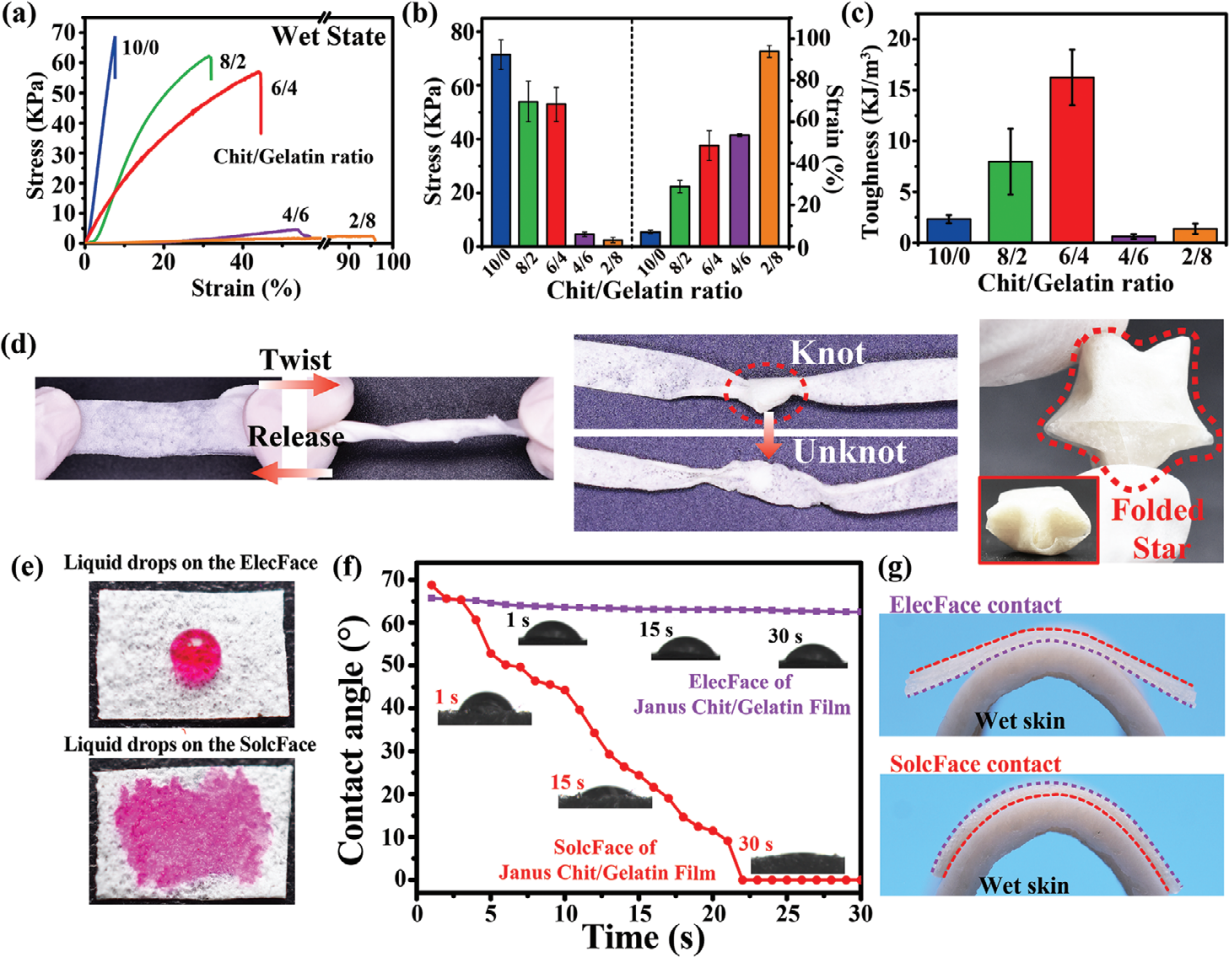
The mechanical properties and surface wettability of Janus Chit/Gelatin films. a–c) Stress‐strain curves and quantitative analysis of wet Janus Chit/Gelatin films demonstrate the mechanical properties vary with gelatin content. (n = 4). d) Optical images demonstrate the flexibility of Janus Chit/Gelatin films (6/4; 6.67 mA cm^−2^ for 1000 s): the wet films can be twisted (tied and untied into a knot) and dry films can be folded into a compact star structure. e) Differential wetting by liquid drops for the dense ElecFace or porous SolcFace of the dry Janus Chit/Gelatin film. f) Dynamic water contact angle measurements show the porous SolcFace promotes liquid spreading while the dense ElecFace appears hydrophobic. g) The dense ElecFace and porous SolcFace of the dry Janus film adhere differently to the surface of wet pigskin. (Note: none of the samples characterized above have been chemically crosslinked).

The most obvious benefit of biology combining partial proteins and polysaccharides and controlling their spatial distribution is optimized mechanics. For instance, the compositional gradients of polysaccharides (i.e., chitin) and proteins in the cuticle of spider teeth and squid beaks that provide both strength for preying and flexibility to reduce self‐injury.^[^ ^1^, ^25^ ^]^ We hypothesis one potential advantage of the two‐component Janus film of chit/gelatin over the single‐component film of chitosan is that it can integrate the strength that mainly conferred by the chitosan‐dense layer and flexibility that conferred by the inclusion of gelatin layer, which would facilitate its manipulation in complex applications (e.g., in a surgical setting). To test this assumption, we synthesized films using various Chit/Gelatin ratios (1% total biomacromolecules; 6.67 mA cm^−2^ for 1000 s), and then measured the mechanical properties of these films under dry and wet conditions. **Figure** **3**a shows stress strain curves for the wet films, while Figure 3b summarizes the results in terms of tensile strength and strain at failure (mechanical property measurements for dried films are shown in Figure S3 in Supporting Information). As can be seen, compared with a single component of chitosan film (i.e., chit/gelatin is 10/0), Janus films containing gelatin components exhibit varying degrees of decrease in tensile strength as the increasing of the gelatin content (presumably it may be due to a slight decline in the chitosan‐enriched dense layer), but significant improvement its abilities to be deformed. Especially, we observe that the Janus film of chit/gelatin electro‐assembled from the mixture with the chit/gelatin ratio is 8/2 or 4/6 can retain ≈80% of the strength of the chitosan film, and the ductility can be improved by nearly 6 times. As shown in Figure 3c, these changes are further quantified by toughness, and Janus film obtained when the ratio of chitosan/gelatin reaches 6/4 shows the highest toughness.

The images in Figure 3d illustrate the flexibility of the Janus chitosan/gelatin film (6/4; electro‐assembled using 6.67 mA /cm^2^ for 1000 s) onto a 10 × 10 cm Ti foil and cut into rectangular shapes. The first image shows the wet film (1 × 3 cm) can be twisted by 720° and upon release returns to its original state. The second image shows a film (0.5 × 6 cm) can be tied into a knot and untied without tearing. The third image shows the film (1 × 10 cm) can be folded into a compact structure (i.e., star). These images illustrate the relative ease for handling the Janus Chit/gelatin film.

The differences between the two faces of the Janus film in compositional and microstructural features result in difference in properties as illustrated by measurements of wettability. In this study, water droplets (20 µL, containing 1 mM Rhodamine) were placed on the different surfaces of Janus Chit/Gelatin film (6/4). As illustrated by the photographs in Figure 3e, drops placed on the porous SolcFace surface were completely absorbed, while drops places on the dense ElecFace were retained on the surface. A more quantitative analysis is illustrated in Figure 3f which shows a rapid decrease in the contact angle for a drop placed on the porous SolcFace while the contact angle for the dense ElecFace remains relatively constant over the 30 sec measurement time (note: both the dense ElecFace and SolcFace of electro‐assembled chitosan control film showed a stable contact angle of about 70° as shown in Figure S2c, Supporting Information). One consequence of the property differences between the two faces of the Janus Chit/Gelatin film is that they exhibit different conformal properties when contacted with wet pigskin surfaces as illustrated in Figure 3g, and this features makes it easy to distinguish the different faces of Janus films in practical applications.

### 2.5 Electronic Tuning of Microstructure and Resultant Properties of Janus Film

As noted, electrodes can impose precise electronic inputs that provide both the chemical and electrical cues for film fabrication. The chemical (i.e., pH) cue is controlled by the electrical current which is directly proportional to the rate of hydroxide ion generation, and the rates of chitosan's deprotonation and gel‐formation. The electric field cue is controlled by the imposed electrode potential but also depends on solution conditions (e.g., salt screening). Importantly, while the electrical current and potential cannot be independently controlled, but generally the change in electrical current and potential is consistent (i.e., a higher electrical current corresponds to a higher electric potential). Here we typically perform experiments using a controlled current density. In subsequent experiments were performed electro‐assembly at different, constant current densities (ranging from 1.67 mA cm^−2^ to 13.33 mA cm^−2^ for 1000 s) using Ti foil electrodes (2 cm × 2 cm) and a electro‐assembly solution containing 1% w/v biomacromolecules (chitosan/gelatin ratio 6/4; pH = 4.5). As expected, the measured electric potential is higher when electro‐assembly is performed at higher current densities (Figure S4d in Supporting Information).

**Figure 4:**
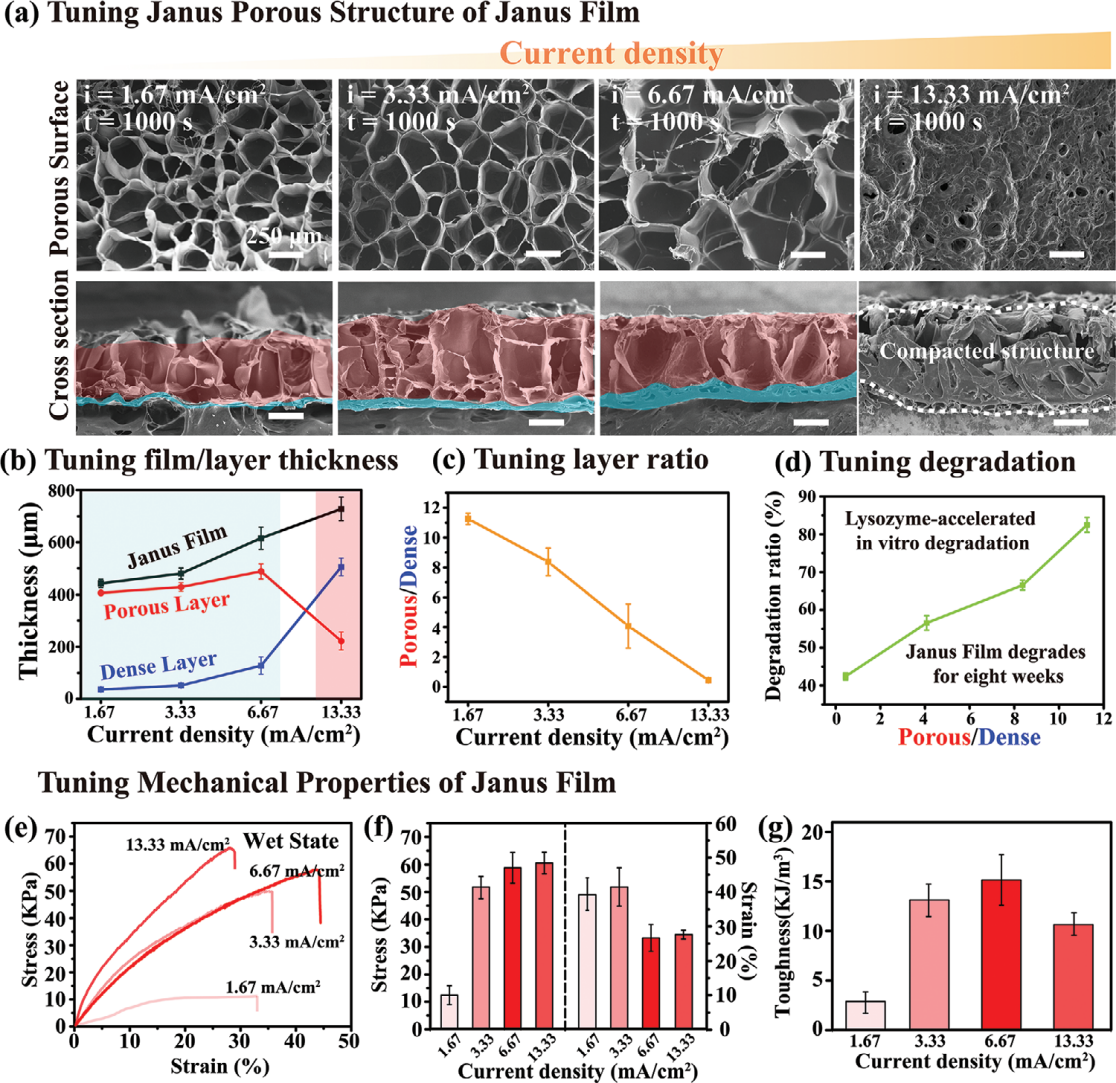
Tuning the microstructure, degradation and mechanical properties of the Janus Chit/Gelatin film by the imposed current density. a) SEM images of the porous surface and cross‐section indicate that a Janus microstructure emerges when a film is electro‐assembled from a chitosan/gelatin solution (Chit/Gelatin is 6/4; 1000 s) within a current density range of 1.67 to 6.67 mA cm^−2^ (the dense layer is marked by a pseudo‐blue color and the porous layer marked by a pseudo‐red color). b) The thickness of the dense layer in the Janus Chit/Gelatin film increases with current density but at a high current density of (i.e., 13.33 mA cm^‐2^) the formation of the porous layer is disrupted. (n = 6). c) The thickness ratio of Porous/Dense layers is tuned by the imposed current density (Chit/Gelatin is 6/4, 1000 s). (Note: none of the samples characterized above have been chemically crosslinked). (n = 6). d) The in vitro enzymatic degradation ratio of the chemically crosslinked Janus Chit/Gelatin film varies with microstructure (5000 U mL^−1^ lysozyme in PBS; 37 °C; 8 weeks; n = 6). e) Mechanical testing of wet uncrosslinked Janus Chit/Gelatin films fabricated using different current densities along with the quantitative summary of f) ultimate stress and strain, and g) toughness (n = 4).

The first row of **Figure** **4**a displays representative scanning electron microscopy (SEM) images of the SolcFace surface of the Janus Chit/Gelatin films prepared at various current densities after freeze‐drying. The results demonstrate that as the current density increases from 1.67 mA cm^−2^ to 6.67 mA cm^−2^, the pore size of the Janus Chit/Gelatin film's SolcFace remains similar. However, when the current density was increased to 13.33 mA cm^−2^, a non‐porous and uneven surface was generated.

The cross‐sectional SEM images in the second row of Figure 4a reveal more internal details of the Janus Chit/Gelatin films prepared at different current densities. With increasing current density, the thickness of the film progressively increases, while the proportion of the dense layer thickness also increases (indicated by the area marked with pseudo‐blue color). At the highest current density of 13.33 mA cm^−2^, the porous layer of the Janus Chit/Gelatin film is not observed but rather the film appears dense throughout. A quantification of these SEM images is summarized in Figure 4b, while Figure 4c shows that the relative thicknesses of the dense and porous layers can be tuned by the current densities. Presumably, high electrical current (i.e., high electrical potential) provides a greater electrostatic force to drive anionic gelatin chains near/inside the assembled film away from the region close to electrode, so that the dense layer of the assembled Janus film increases while the porous layer that containing gelatin decreases.

In certain biomedical applications, Janus Chit/Gelatin films may require chemical crosslinking to extend their service life in vivo. Figure S6 of Supporting Information shows that the crosslinked Janus Chit/Gelatin films still retain their unique Janus porous structure and related structural characteristics (e.g., porosity and film thickness), without substantial structural loss due to crosslinking (e.g., densification of the gel network). We speculate that this is primarily because the chitosan chains in the film form a relatively stable primary physical crosslink (i.e., crystal network junction) after deprotonation as illustrated in Scheme 1a.

One advantage of using biomacromolecules (i.e., chitosan and gelatin) is that the resulting materials should be biodegradable although the microstructure is expected to affect the rate of biodegradation. This is illustrated by an in vitro enzymatic biodegradation study in which Janus Chit/Gelatin films (6/4) prepared at different current densities (note: all samples crosslinked by 0.5% w/v glutaraldehyde for 30 min) were incubated with a chitosan‐degrading enzyme lysozyme (5000 U/mL lysozyme in PBS buffer, pH 7.4). As expected, Figure 4d shows that Janus films prepared at lower current densities (with a larger proportion of porous to dense layer) was observed to undergo greater degradation after 8 weeks of incubation (Figure S4e in Supporting Information shows the time‐dependent degradation ratio). It should be noted that the degradation of two layers in the Janus film doesn't seem to be synchronized. After 8 weeks of degradation, the chitosan dense layer of Janus film still maintains its structural integrity, while the chitosan/gelatin porous layer has obvious collapse and damage of the pores, as shown in Figure S4f (Supporting Information).

Differences in the Janus film's microstructure (generated by electro‐assembly at different current densities) are also expected to alter the film's mechanical properties. Figures 4e–g show the stress‐strain curves for wet films prepared at different current densities and the quantitative summary of their mechanical properties. Overall, the results show that films prepared at higher current densities tended to be stronger (higher tensile strength) but more brittle (lower strain at failure). The Janus Chit/Gelatin film prepared at a current density of 6.67 mA cm^−2^ exhibits the highest toughness. Besides, wet Chit/Gelatin Janus films prepared under the conditions of different current densities also show stable mechanical properties with no damage, mechanical degradation or irreversible deformation under dynamic loading (Figure S5, Supporting Information)

Overall, the result in Figure 4 illustrate that the electronic inputs can be controlled to “tune” microstructural features that significantly affect mechanical and biological (i.e., biodegradation) properties.

### 2.6 Directional Transport Properties of Janus Film

**Figure 5:**
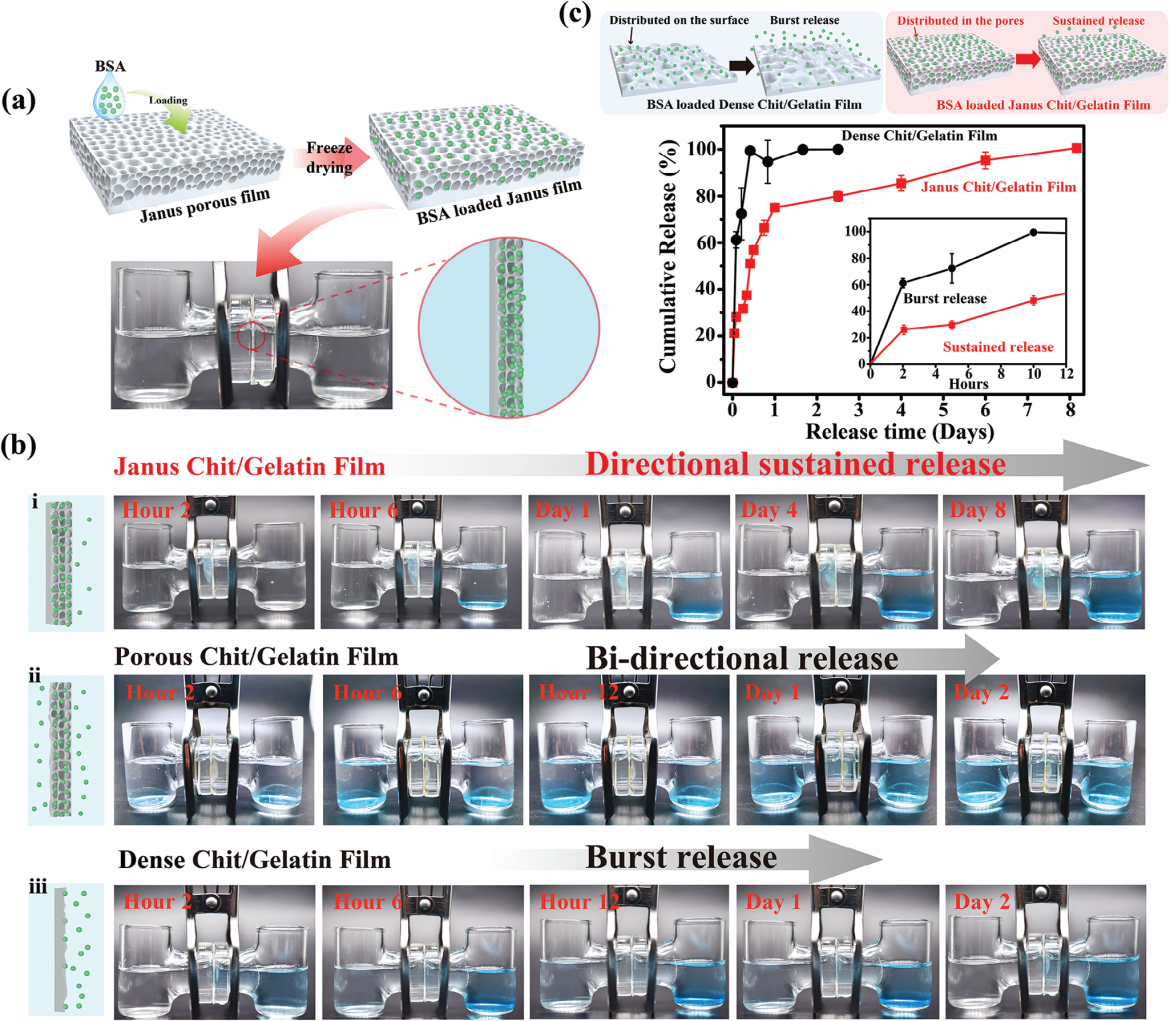
Janus microstructure confers directionality to molecular transport. a) Illustration of the loading of the model protein, bovine serum albumin (BSA), into the porous layer of a Janus Chit/Gelatin film and measurement of its direction‐dependent release. b) Images of BSA release into the two compartments of the diffusion cell for: i) the Janus Chit/Gelatin film; ii) a porous Chit/Gelatin control film prepared by casting and freeze drying; and iii) a dense Chit/Gelatin control film prepared by casting (note: Coomassie Brilliant Blue G250 was added to the solution in the two chambers to facilitate observation). c) Real‐time observation of BSA release from films into the solution of the two chambers (n = 4), and the schematic above shows that BSA is mainly distributed on the surface of dense Chit/Gelatin film and distributed in the pores of Janus Chit/Gelatin film, resulting in their different release. (Note: all the samples characterized above have been chemically crosslinked by 0.5%w/v glutaraldehyde for 30 min).

Because of its asymmetric microstructure, the Janus Chit/Gelatin film is expected to have different transport properties between the two layers. To test this expectation, **Figure** **5**a shows that we: prepared a Janus Chit/Gelatin (6/4) film (note: all groups of film were cross‐linked with 0.5% w/v glutaraldehyde); loaded the porous face with the model protein bovine serum (BSA) and freeze dried the film; sealed and fixed the film in the diffusion cell to separate the two chambers; added 5 mL PBS (pH = 7.4) to each of the two chambers; and then measured the release of the BSA from the film. To visualize and quantify BSA release, we added Coomassie Brilliant Blue G‐250 to each chamber: this marker combines with BSA to generate a blue color that depends on BSA concentration. Figure 5b(i) shows that over the course of 8 days, the BSA was observed in the right chamber but not the left chamber. This result shows that the BSA can be released from the porous layer of the Janus chitosan/gelatin film while the dense layer serves as a barrier for the transport of BSA.

For comparison, we prepared two control chitosan/gelatin films by casting (i.e., not by electro‐assembly). The first control was a “porous chitosan/gelatin” film that was prepared by casting a chitosan/gelatin solution, partially drying and neutralizing the film, and then freeze drying the film to generate a porous structure (porosity of ≈76%) that was comparable to the porosity to the porous layer of the Janus Chit/Gelatin film. The BSA was then loaded throughout this porous film and the film was again freeze dried before testing. As expected, Figure 5b(ii) shows that BSA was released from this porous film into both chambers of the diffusion cell and thus there was no preferential direction for release.

The second control was a “dense chitosan/gelatin” film that was prepared by casting a chitosan/gelatin solution, fully drying and neutralizing the film to generate a film of thickness (≈150 µm) similar to that of the Janus Chit/Gelatin film's dense layer. The BSA was then applied to one face of this dense film and freeze dried before testing. Figure 5b(iii) shows that the BSA appears in the right chamber but not the left chamber indicating that the BSA cannot readily pass through the dense layer for release into the left chamber.

While both the Janus Chit/Gelatin film and the control dense film show directional release of the BSA, Figure 5c shows the release from the dense control film is more rapid. Presumably, dense Chit/Gelatin film has a dense non‐porous structure, the loaded BSA is more distributed on the surface of the film thus led to such rapid release, while the microstructure of porous layer of the Janus Chit/Gelatin film impedes the release of the BSA by providing more surface area for BSA adsorption.^[^ ^26^ ^]^ In many applications (e.g., controlled drug release), a slower, more sustained release is beneficial compared to a rapid burst pattern of release.

### 2.7 Multiple Biological Functions of Janus Film

**Figure 6:**
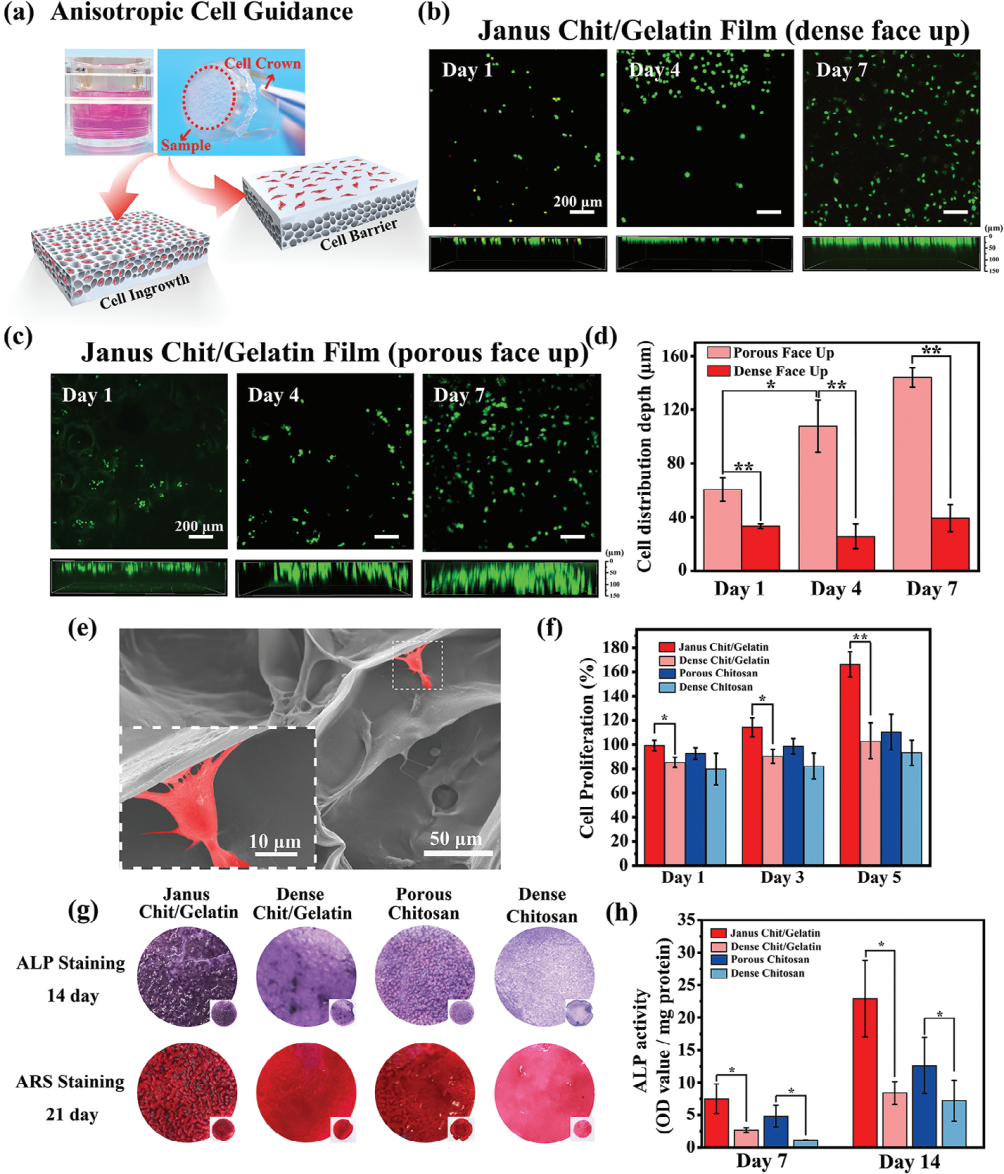
Janus features prevents fibroblast infiltration and promotes osteoblast proliferation and differentiation. a) Experimental approach in which cell crown insert is sealed with a crosslinked film (dense face up or porous face up) and placed on a well plate to isolate upper and lower volumes to assess anisotropic cell guidance. b–d) 3D fluorescence images show that L929 fibroblast cells did not penetrate through the dense layer but grew into the porous layer (n = 4). e) SEM images of MC3T3‐E1 osteogenic precursor cells (marked by pseudo‐red color) show cell adhesion in the porous layer of the Janus Chit/Gelatin film. f) Comparison of the proliferation of osteogenic precursor cells (MC3T3‐E1) on different films suggests both the inclusion of gelatin and the Janus microstructure promote cell proliferation (n = 4). g) ALP and Alizarin Red S staining of cells seeded on the surface of different films indicate that the Janus Chit/Gelatin film promotes osteogenic differentiation. h) ALP activity of MC3T3‐E1 cells seeded on the surface of different films further indicate the composition and microstructure of Janus Chit/Gelatin film are important for promoting osteoblast differentiation (n = 4). (*p < 0.05, **p < 0.01).

The integration of microstructural and compositional features in a Janus film enables better customization of its local physicochemical properties for tailoring cellular behavior and conferring specific biological functions. One illustrative case is guide bone regeneration (GBR), and the Janus film must perform two essential functions for controlling cell responses. First, the dense layer must perform a barrier function to prevent the invasion of connective tissue cells into bone defects. To test this function, we evaluated whether L929 fibroblasts could penetrate the two surfaces of Janus Chit/Gelatin film (6.67 mA cm^−2^, 1000 s, dense layer ≈150 µm, porous layer ≈400 µm, and stabilized the film by cross‐linked with 0.5% w/v glutaraldehyde). These experiments were carried out using the system shown in **Figure** **6**a, where the cell crown inserts were sealed with Janus Chit/Gelatin films (either dense side facing up or porous side facing up) and placed on well plates to separate the two volumes. The fibroblast cells were then inoculated in the upper volume, and their distribution in Janus Chit/Gelatin film was assessed after various incubation times. For analysis, live/dead staining of cells at different time points was performed, and the live cells were assessed using confocal laser microscopy (green staining).

Fluorescent images in Figure 6b depict the fibroblast cell distribution when seeded on the dense surface. The film surface (top image) and optical cross‐section (bottom image) exhibit a consistent increase in live cell count, suggesting that these fibroblasts proliferate throughout the 7‐day incubation period. The fluorescent cross‐sectional images demonstrate that cells are localized within a narrow region adjacent to the dense layer surface of Janus Chit/Gelatin film during the entire culture process. Conversely, Figure 6c reveals that when fibroblasts are seeded on the porous surface, they not only proliferate but also infiltrate the porous surface. To compare the penetration depth of cells on the different surfaces of the Janus Chit/Gelatin film, we analyzed the cross‐sectional fluorescent images (note: because the films are not entirely flat it was difficult to accurately locate the film surface and thus the analysis of penetration depth is semi‐quantitative). Figure 6d illustrates that when fibroblast cells were exposed to the dense surface, they were confined to a narrow region near the surface throughout the 7‐day culture period. In contrast, when the fibroblast cells were exposed to the porous surface, they were observed to penetrate deeper into interior regions of the film. These results demonstrate that the Janus Chit/Chitosan film can perform the first function with the dense film serving as a barrier to fibroblast penetration.

The second biological function that the Janus Chit/Gelatin film must perform is that the porous layer must provide the physicochemical microenvironment necessary to promote the adhesion, proliferation, and differentiation osteoblasts cells. To test this function, we cultured the osteoblast precursor cell line MC3T3‐E1 on the porous surface of Janus Chit/Gelatin film. The SEM images in Figure 6e demonstrate that after just one day of seeding osteoblast cells were observed to adhere onto the porous surface of the Janus Chit/Gelatin film. Further SEM images taken at higher magnification reveal excellent cell spreading, characterized by filamentous pseudopodia and unidirectional lamellipodia (marked by pseudo‐red color).

The underlying assumption of this work is that both composition and microstructure are important to this second biological function. To assess this assumption, we compared the ability of various films to support osteoblast cell proliferation: (i) the “Janus Chit/Gelatin” film; (ii) a “Dense Chit/Gelatin” film prepared by casting as described in Figure 5; (iii) a “Porous Chitosan” film prepared by a solution freeze‐drying method which had a similar porosity (≈76%) to that of the Janus Chit/Gelatin film; and (iv) a “Dense Chitosan” film prepared by electro‐assembly. Experiments were performed by seeding 5 × 10^4^ osteoblast cells per well on different film surfaces, culturing for varying times, and measuring proliferation using the Cell Counting Kit‐8 (CCK‐8, Dojindo). Figure 6f compares the osteoblast proliferation for these different films at different time points [note: 100% is normalized to proliferation observed on tissue culture polystyrene plate (TCP) at the same day as the measurement]. We observed that all films supported cell proliferation, although the proliferation on the dense films were lower than proliferation observed on TCP. Most importantly, Figure 6f shows that significantly higher osteoblast proliferation was observed for the Janus Chit/Gelatin film compared to any of the control films and TCP. These results support our assumption that a porous microstructure and the inclusion of gelatin into the porous layer would enhance the film's biological function for our GBR application.

We next compared the in vitro osteogenic differentiation potential of these four films by seeding osteoblast cells, and after one day replacing the culture medium with an osteogenic induction medium. Osteogenic markers were subsequently measured at different time points. Alkaline phosphatase (ALP), one such marker, was evaluated through staining after 14 days of incubation. The top panel of Figure 6g shows that the Janus Chit/Gelatin films exhibited visually higher ALP staining compared to control films. A second osteogenic marker, calcium mineral, was measured using Alizarin Red S (ARS) staining. After 21 days of incubation, the films underwent ARS staining, the results of which are depicted in the bottom panel of Figure 6g. The porous layers of the Janus Chit/Gelatin films showed deeper ARS staining compared to the control films, indicating a greater mineral nodule density on the material surface. The ARS and ALP measurements both indicate greater osteoblast differentiation when cultured on the porous face of the Janus Chit/Gelatin film.

In a separate experiment, we seeded osteoblast cells on the various films and performed quantitative ALP analysis (Beyotime, ALP kit) 7 and 14 days after transfer to the differentiation medium. Figure 6h shows that the Janus Chit/Gelatin had higher ALP activity at both time points indicating that this film promoted osteoblast differentiation better than the control films. This result again supports our assumption that both composition and microstructure are important for conferring biological function. Potentially, the inclusion of gelatin into the film improves biological function by providing molecular cues (e.g., RGD sequences) that enhance cell adhesion.^[^ ^27^ ^]^

In summary, these in vitro cell studies confirm that both the film's composition and microstructure are important for conferring biological functions important for guided bone regeneration. The Janus microstructure is important for both preventing fibroblast invasion while promoting the growth and differentiation of osteoblast cells. The inclusion of gelatin into the porous layer also appears to enhance osteoblast cell growth and differentiation.

### 2.8 In Vivo Tissue Responses and Biodegradation of Janus Films

We next evaluated the effect of microstructure and composition on the in vivo tissue responses and degradation of different films by implanting them subcutaneously on the left and right sides of the dorsal region of 12 rats. Experimentally, we tested Janus Chit/Gelatin films (6/4) prepared at two distinct current densities (3.33 mA cm^−2^ and 6.67 mA cm^−2^) to evaluate different Janus microstructures. Additionally, we evaluated two control films: a “Dense Chitosan” film prepared by electro‐assembly and a “Dense Chit/Gelatin” film prepared by casting as described in Figure 5. All films were partially stabilized by 0.5% w/v glutaraldehyde crosslinking for 30 min before implantation. Throughout the entire observation period, all mice remained healthy, with no surgery‐related infections or complications observed. Photos were taken, and dorsal skin tissues with integrated materials were collected at 4 and 8 weeks post‐implantation.

**Figure 7:**
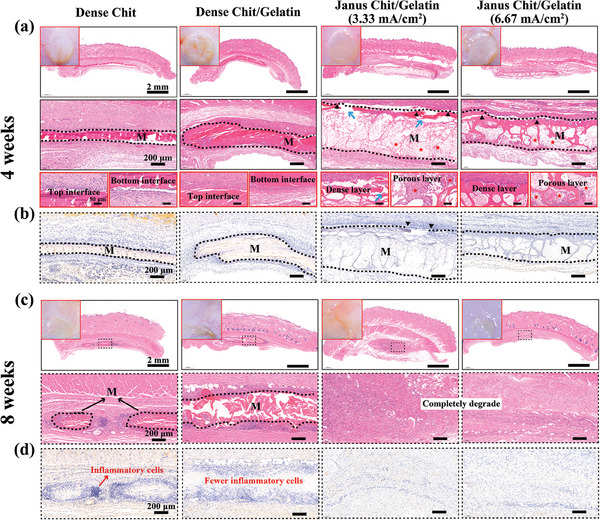
Tissue responses to different films after subcutaneous implantation in rats. a) Optical images, H&E staining images with different magnifications and b) immunohistochemistry (IHC) staining of IL‐6 4 weeks after subcutaneous implantation for control films (dense Chit or dense Chit/Gelatin) and the Janus Chit/Gelatin films electrofabricated under different conditions (3.33 mA cm^−2^ and 6.67 mA cm^−2^). [Note: black dotted line indicates the area of implanted materials, M; black triangle marks the dense layer of Janus film; red star marks the tissue ingrowth into the Janus porous film; and blue arrows mark the broken dense layer of Janus Chit/Gelatin (3.33 mA cm^−2^)]. c) H&E staining images with different magnifications and d) IL‐6 IHC staining for the different film treatments 8 weeks after subcutaneous implantation. (Note: All the films mentioned above were chemically crosslinked using 0.5%w/v glutaraldehyde for 30 min).

The top two rows of **Figure** **7**a show the macroscopic photos (top‐left inset) and histological H&E‐stained tissue sections at low and high magnification, taken 4 weeks after material implantation. In all cases, the sites of implantation appeared healthy with no noticeable redness or swelling. The low magnification images in the top row of Figure 7a show all the implanted films (designated “M”) remained intact and integrated well with the tissue (i.e., no separation from the upper skin tissue was observed). The higher‐magnification histological H&E‐stained images in the second row reveal that the films from the various groups essentially maintained their structure after 4 weeks implantation. For both Janus Chit/Gelatin film groups, the distinct Janus porous structure is visible (dense layer facing up, and porous layer facing down). Further, these images show the Janus Chit/Gelatin film (6.67 mA cm^−2^) with the thicker dense layer (marked with black triangles) remained intact without significant degradation or damage after 4 weeks of implantation. The Janus Chit/Gelatin (3.33 mA cm^‐2^) with the thinner dense layer exhibited localized degradation and damage (marked with blue arrows).

The third row of Figure 7a is the highest‐magnification of histological H&E‐stained images and shows the histological response of the top and bottom surfaces of different films to the tissue contact. The Dense Chit and Dense Chit/Gelatin films groups at the left show fibrous tissue could only grow along the film's top or bottom surfaces without penetrating the film's interior, which indicates that these dense films can serve as a physical barrier to fibrous tissue penetration. For the Janus Chit/Gelatin (6.67 mA cm^−2^) film, the third‐row image on the left shows fibrous tissue growth along the surface of the dense layer with no obvious penetration into the film. For the dense layer of Janus Chit/Gelatin (3.33 mA cm^−2^) film, some fibroblasts were observed to penetrate through a partially degraded region (marked with blue arrow) indicating that the dense layer of this Janus Chit/Gelatin film was not sufficiently thick to resist degradation within the first 4 weeks. The images of the porous layers at the right in the third row for both Janus Chit/Gelatin films show cellular ingrowth (marked with red asterisk), which indicates that the porous structure can provide the necessary 3D space for cell recruitment.

The Immunohistochemical stain of IL‐6 shown in Figure 7b further revealed that there was no excessive inflammation 4 weeks post‐implantation in the different film groups. (note: the Immunohistochemical stain of TNF‐α was shown in Figure S8 in Supporting Information)

Figure 7c shows the macroscopic photos (top‐left inset) and histological H&E‐stained tissue sections 8 weeks after implantation under the skin. The macroscopic photos (top‐left inset) show that after 8 weeks, subcutaneous material is no longer apparent. The high‐magnification H&E‐stained images reveal that films in various groups experienced different degrees of degradation after 8 weeks of implantation. For the two dense films (Dense Chit film and Dense Chit/Gelatin), remnants of the degraded films were still discernible (surrounded with black dotted line) 8 weeks post‐implantation. In contrast, remnants of the two Janus Chit/Gelatin films were not observed, indicating these films were completely degraded.

The immunohistochemical results in Figure 7d showed that after material degradation, the two groups implanted with Janus Chit/Gelatin films did not exhibit high expression of inflammation‐related IL‐6 or infiltration of associated inflammatory cells. However, the group implanted with dense chit films displayed noticeable infiltration of blue inflammatory cells around the materials and high expression of IL‐6. Interestingly, the dense Chit/Gelatin film, although also not completely degraded, had fewer blue inflammatory cells around the material and relatively lower expression of IL‐6. Presumably, the lower inflammation in the dense Chit/Gelatin film implanted group may be related to the gelatin composition in the film. Specifically, chitosan is not native in humans, and possibly, the biocompatibility is improved by incorporating gelatin which is derived from collagen which is abundant in the ECM.

In summary, the Janus films demonstrated excellent biocompatibility and biodegradability in these subcutaneous implantation studies. In particular, the Janus Chit/Gelatin films (6.67 mA cm^−2^) maintained its structural integrity for at least 4 weeks post‐implantation, but was completely degraded within 8 weeks without inducing excessive inflammatory reactions. Further, the anisotropic microstructure of the Janus Chit/Gelatin films appears to perform both functions with the dense layer serving as a barrier against fibrous tissue invasion, while the porous layer serving to promote cells ingrowth. For GBR film, it is generally necessary to ensure that at least 4 weeks can act as a barrier to fibroblasts.^[^ ^28^ ^]^ In cases when a longer barrier period is required, this could presumably be achieved by adjusting the process parameters to increase the thickness of the dense layer in the film or optimizing the cross‐linking conditions to prolong the degradation of entire film.

### 2.9 In Vivo Studies of Guided Bone Regeneration

**Figure 8:**
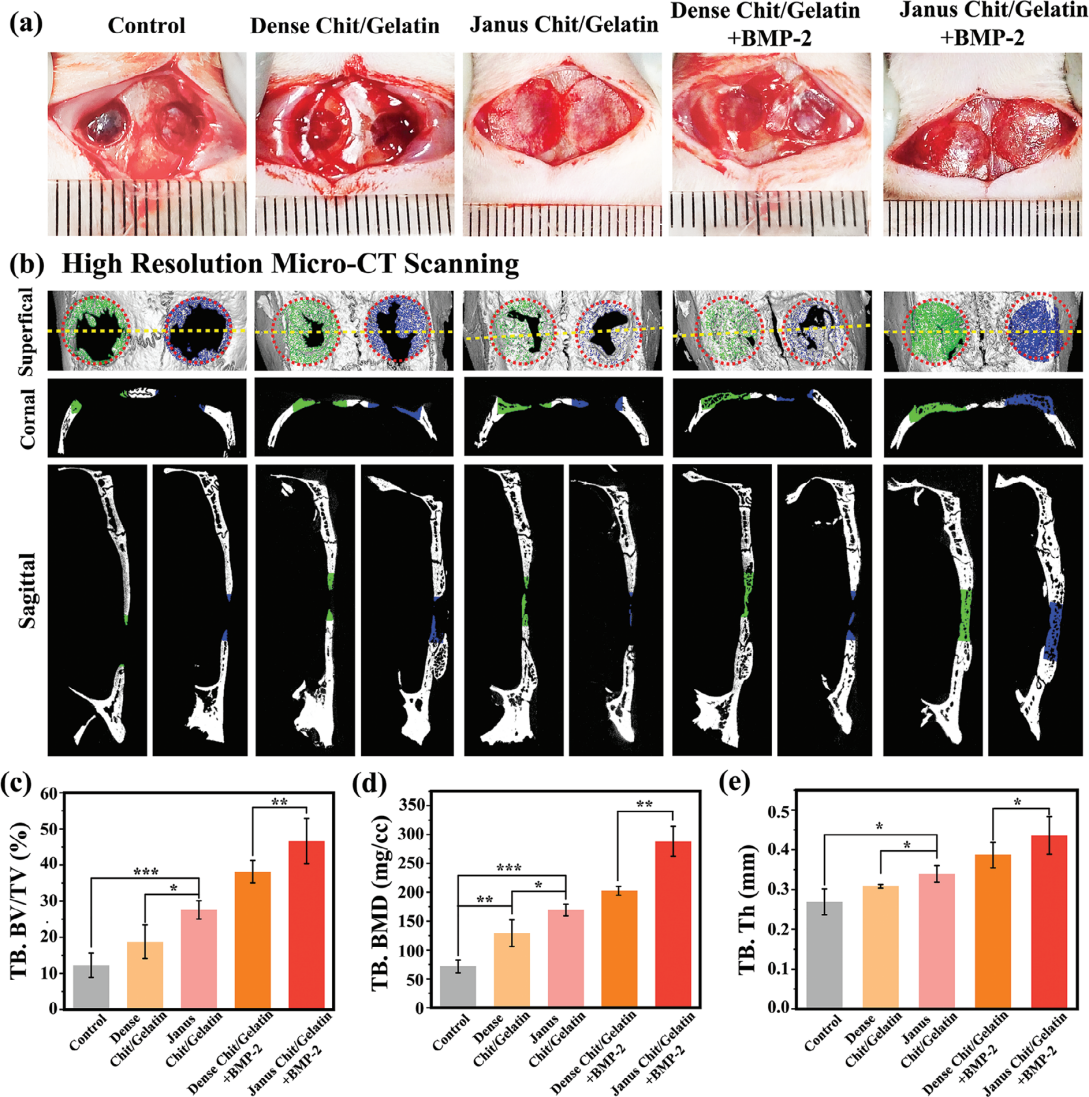
Evaluation of Janus Chit/Gelatin film for guided bone regeneration in a cranial defect model of rats. a) Illustrative images show surgical placement of films on rat calvarial defects in different groups and no‐film placed on the defects of control groups. (Note: All the films mentioned above were chemically crosslinked using 0.5%w/v glutaraldehyde for 30 min). b) Micro‐CT imaging of treated calvarial defects taken at 8 weeks after surgery; c) Trabecular bone volume to tissue volume (Tb. BV/TV); d) Trabecular bone mineral density (Tb. BMD) and e) trabecular thickness (Tb. Th) evaluated by morphometric analysis. (*p < 0.05, **p < 0.01, ***p < 0.001; n  =  6).

Finally, the in vivo osteogenic potential of the Janus Chit/Gelatin film for guided bone regeneration was assessed using a cranial defect model in 15 rats. In this study, two identical critical‐sized defects (5 mm) were generated in the animals' skulls (these defects are too large to heal spontaneously during the animal's lifetime).^[^ ^29^ ^]^ After creating these defects, various films were implanted to cover each of the defects, and outcomes were compared to a no film treated “control” group, as illustrated in **Figure** **8**a. We selected the Janus Chit/Gelatin film (6.67 mA cm^−2^) as our test film due to its long‐term barrier functionality (conferred by the dense layer; Figure 7) and outstanding osteogenic activity (conferred by the porous layer; Figure 6). As a control film, we tested a Dense Chit/Gelatin film prepared by casting as described in Figure 5. All films were partially stabilized by 0.5% w/v glutaraldehyde crosslinking for 30 min before using. In clinical practice, a common approach to accelerate bone regeneration is to incorporate bone morphogenetic protein‐2 (BMP‐2) into implanted GBR films.^[^ ^30^ ^]^ Ideally, BMP‐2 release should be sustained and directionally‐controlled to promote therapeutic efficacy and avoid ectopic ossification. Thus, we also tested Chit/Janus films with dense and Janus microstructures that had been loaded with BMP‐2 (5 µg per film using the loading method is described in Figure 5).

Eight weeks after surgery, the animals were euthanized and high‐resolution micro‐CT scans were measured to observe new bone formation as shown by the representative images in Figure 8b. New bone formation (marked by pseudo‐green and pseudo‐blue color for the left and right sides, respectively) was observed in all groups, but with varying degrees among the five groups. The Chit/Gelatin groups that lacked BMP‐2 showed more newly‐formed bone tissue (compared to the no film “control” group), while the Janus Chit/Gelatin group showed somewhat more new‐bone formation (compared to the Dense Chit/Gelatin group). The two Chit/Gelatin groups in which the films were loaded with BMP‐2 showed significantly more newly‐formed bone tissue (compared to the groups without BMP‐2. The defects on both left and right sides of the Janus Chit/Gelatin film with BMP‐2 loading were almost entirely restored, with thicker and denser new bone tissue visible in both sagittal and coronal positions. In contrast, the right side (indicated in blue) of the defects covered by the Dense Chit/Gelatin film with BMP‐2 loading still had partial defects, with thinner and more sparse new‐bone tissue visible in both sagittal and coronal positions.

Quantitative morphological analyses using micro‐CT summarizes the results for GBR in terms of trabecular bone volume to tissue volume (Tb. BV/TV; Figure 8c), Trabecular bone mineral density (Tb. BMD; Figure 8d) and trabecular thickness (Tb. Th; Figure 8e). As shown, 8 weeks after implantation, all morphological indices demonstrate the benefits of: using a film (vs no film “control”); films with a Janus microstructure (vs only a dense structure); and loading of BMP‐2 into the films. Presumably, the superior performance of the BMP‐2‐loaded Janus Chit/Gelatin film (vs the BMP‐2‐loaded Dense Chit/Gelatin film) reflects the capability of the porous layer to both allow sustained and directional release of BMP‐2 as well as promote the recruitment of osteoblast cells.

**Figure 9:**
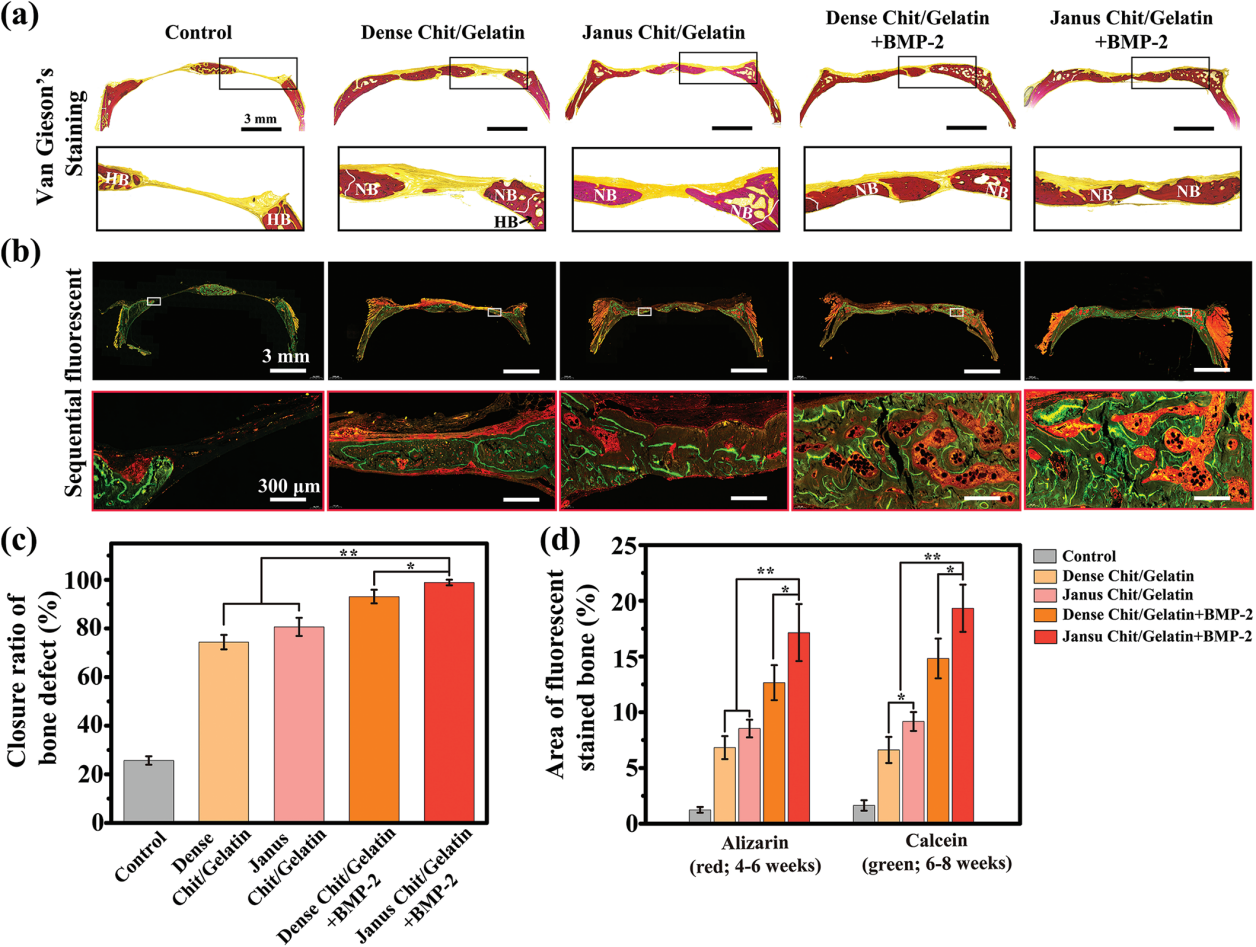
Histological analysis of bone defects treated with Janus Chit/Gelatin films. a) Van Gieson's picrofuchsin of a newly formed bone in different groups after 8 weeks repair period. b) Fluorescent‐labeling histomorphometrical analysis of new bone formation and mineralization at 8 weeks post‐operation (red, between 4 and 6 weeks; green, between 6 and 8 weeks). Semi‐quantitative ratio of c) the closed bone defect (%) by the Van Gieson's picrofuchsin slice and d) the stained bone area.(*p < 0.05, **p < 0.01, ***p < 0.001; n  =  6).

We also used a standard histological method of Van Gieson staining to intuitively observe the newly formed bone tissue after 8 weeks operation. The representative histological images in **Figure** **9**a show the fibrous tissue in yellow and bone tissue in dark red, while the enlarged images at the bottom distinguish the host bone tissue (labeled HB) from the newly formed bone tissue (labeled NB). For all groups in which a film was used at the cranial defect site (compared to the no film “control”), more newly‐formed bone was observed with smaller gaps in bone defects. BMP‐2‐loading markedly improved healing with the BMP‐2‐loaded Janus Chit/Gelatin film showing almost complete closure of the defect. Image analysis of these histological sections (Image Pro 5.0, Media Cybernetic, Silver Springs, MD, USA) was used for semi‐quantitative analysis of the bone defect closure rate (i.e., the percentage of the width of newly formed bone relative to the initial 5 mm defect width). Consistent with the micro‐CT results, Figure 9c shows the Janus Chit/Gelatin film effectively guided new bone formation, while the BMP‐2‐loaded Janus Chit/Gelatin film showed the best bone regeneration, successfully achieving full closure of the 5 mm bone defects within eight weeks.

Sequential fluorescent labeling is a technique utilized for marking mineralized tissues and evaluating the dynamics of new bone mineralization during the bone regeneration process. In our study, alizarin red (red) and calcein (green) were intraperitoneally injected into each rat at postoperative weeks 4 and 6, respectively, and the animals were sacrificed at 8 weeks and the cranial bone was sectioned. The fluorescence of these bone specimens was observed using confocal laser microscopy with the total fluorescence (red plus green) indicating the total calcified bone with the green fluorescence indicating the most recently formed calcified bone. The fluorescence images in Figure 9b indicate that more calcified bone was observed at the cranial defect site for groups with: (i) a film (compared to the no film “control”); (ii) a film with a Janus microstructure (compared to films with only a dense microstructure); and (iii) with BMP‐2‐loading of the films. These results are consistent with the micro‐CT results and further suggest that the Janus microstructure enhances the effects of BMP‐2‐loading possibly by controlling its release. The image analysis in Figure 9d provides a semi‐quantitative summary of these results by showing the percentage of the imaged area that had red or green fluorescence. In summary, these histological results are consistent with the micro‐CT results (Figure 8), indicate that the Janus Chit/Gelatin film with porous Janus structure can effectively guide bone regeneration and enhance the effect of BMP‐2 loading.

## 3 Conclusion and Outlook

We report the electrofabrication of a Janus film from gelatin and chitosan to meet the diverse and demanding requirements for guided bone regeneration (GBR). By using well‐established biomedical biomacromolecules, our electrofabricated films are intrinsically biodegradable and biocompatible. Further, the compositional and microstructural gradients of the Janus film confers asymmetric properties important for the GBR application. We show that the chitosan enriched dense face serves as a barrier for molecular transport and cell infiltration, while the porous face containing chitosan and gelatin allows transport and promotes osteoblast ingrowth and differentiation. Finally, we used a rat cranial defect model, and observed that the electrofabricated Janus Chitosan/Gelatin film offers superior performance for bone regeneration, especially when combined with the BMP‐2 growth factor. Compared to previously‐reported single‐component (i.e., chitosan) Janus porous films, this compositional and microstructural gradient integrated two‐component (i.e., chitosan and gelatin) Janus film shows enhanced flexibility and anisotropic biological multi‐functions, making it easier to handle in a surgical setting and better able to guide bone regeneration by enhancing osteoblast proliferation and differentiation.

Electrofabrication is an emerging additive manufacturing method that is simple, rapid (<20 Min) and safe (<10 V), and offers unprecedented capabilities for controlling microstructure. In our studies, we use electronic inputs to impose the chemical (i.e., pH) and electrical cues that induce the bottom‐up self‐assembly of chitosan and control its emergent structure. We show that when electro‐assembly is performed from a mixture of chitosan and gelatin, a film is formed with a gradient in composition consistent with a proposed electro‐molecular sorting mechanism. Also, we show that the electronic inputs can be “tuned” to control important microstructural features of the Janus film (e.g., pore size and porosity of the porous layer, and the ratio of dense to porous layers). Overall, we believe this demonstration of the creation of controlled gradients in composition and microstructure for biomacromolecules provides additional evidence for the versatility of electrofabrication for the customized manufacturing of materials that can perform multiple functions.

Looking forward, we envision considerable opportunities for improving electrofabrication. Specifically, hydrogel structure emerges from complex, dynamically‐changing set of molecular‐level processes that are currently incompletely‐understood. Figure 2a illustrates that far from the electrode, isolated cationic gelatin and chitosan chains are influenced by the electric field to migrate toward the cathode. However, the imposed electric field has additional, more‐subtle effects that can organize the solution's hierarchical structure. Modeling and experiment both indicate that the field can alter chitosan's chain conformation (extended versus collapsed),^[^ ^31^ ^]^ and orientation relative to the electrode (e.g., parallel vs perpendicular)^[^ ^14^, ^32^ ^]^ and possibly even the alignment towards neighboring chains. Attenuating these electrostatic forces (e.g., through salt addition) has been shown to dramatically affect the hydrogel's micro‐structure and functional properties.^[^ ^22^, ^24^, ^33^ ^]^ Near the assembly front, Figure 2a illustrates that there is a steep pH gradient.^[^ ^20^ ^]^ In essence, the electrode serves as a sink of protons that are stripped from the gelatin and chitosan chains, and this change in protonation state alters the balance of inter‐polymer interactions by diminishing electrostatic repulsions and enabling attractive mechanisms to promote associations between polymer chains. For chitosan, experimental^[^ ^34^ ^]^ and modeling^[^ ^14b^ ^]^ studies indicate that chitosan's self‐assembly involves the formation of crystalline network junctions that are stabilized by the localized hydrophobic region within the crystallite (i.e., the pKa of chitosan's amino groups is lowered).^[^ ^35^ ^]^ Here, we show that at intermediate pHs (that occur near the assembly front), gelatin and chitosan can undergo polyelectrolyte complexation although the microstructure and stability (i.e., lifetime) of these gelatin‐chitosan complexes is unknown. Our results suggest that these gelatin‐chitosan complexes can dis‐associate since gelatin is excluded from the dense layer within the assembly front, yet these complexes appear to be sufficiently stable to be incorporated into the porous layer. It is currently unclear what underlying mechanisms (or combination of mechanisms) are responsible for gelatin's promotion of pore formation (Figure 2) and why accelerating self‐assembly (by increasing the imposed current) diminishes the formation of a porous microstructure (Figure 4). Finally, Figure 2a illustrates that close to the electrode, within the assembly front, chitosan has formed a crystalline hydrogel network. Surprisingly, recent in situ video imaging has shown that this assembled film is not static but can undergo microstructural transitions in the presence of a continued electric field.^[^ ^23^ ^]^ We envision, with greater mechanistic understanding it will be possible to tune the electrical inputs to electrofabricate hydrogels with increasingly complex structures.

## 4 Experimental Section

More details are provided in the Supporting Information.

## Conflict of Interest

The authors declare no conflict of interest.

## Supporting information

Supporting Information

Supporting Information

Supporting Information

## Data Availability

The data that support the findings of this study are available from the corresponding author upon reasonable request.
