# Supplementary material for: Electro‐Sorting Create Heterogeneity: Constructing A Multifunctional Janus Film with Integrated Compositional and Microstructural Gradients for Guided Bone Regeneration
Source: Adv Sci (Weinh). 2024 Jan 15;11(12):2307606. doi: 10.1002/advs.202307606 (PMC10966538; doi:10.1002/advs.202307606)
Supplement: Supplementary file 1 — Supporting Information [file ADVS-11-2307606-s002.pdf]

ADVANCED SCIENCE
Open Access

Supporting Information

for *Adv. Sci.*, DOI 10.1002/advs.202307606

Electro-Sorting Create Heterogeneity: Constructing A Multifunctional Janus Film with Integrated Compositional and Microstructural Gradients for Guided Bone Regeneration

Miao Lei, Haitao Liao, Shijia Wang, Hang Zhou, Jianwei Zhu, Haoran Wan, Gregory F. Payne, Changsheng Liu and Xue Qu**

Supplementary Materials for

Electro-Sorting Create Heterogeneity: Constructing a Multifunctional Janus Film with Integrated Compositional and Microstructural Gradients

Miao Lei [1‡], Haitao Liao [1‡], Shijia, Wang [1], Hang Zhou [1], Jianwei, Zhu [1], Haoran Wan [1], Gregory F. Payne [2,*], Changsheng Liu [1] and Xue Qu [1,3,*]

1 Key Laboratory for Ultrafine Materials of Ministry of Education, Frontiers Science Center for Materiobiology and Dynamic Chemistry, School of materials science and engineering, East China University of Science and Technology, Shanghai 200237 (China)

2 Institute for Bioscience and Biotechnology Research and Robert E. Fischell Biomedical Device Institute, 5118 A. James Clark Hall, College Park, Maryland 20742 (USA)

3 Shanghai Frontier Science Research Base of Optogenetic Techniques for Cell Metabolism, East China University of Science and Technology, Shanghai 200237 (China)

‡ These authors contributed equally to this work.

* Corresponding author emails: gpayne@umd.edu; quxue@ecust.edu.cn

This PDF file includes:

Experimental Section
Figs. S1 to S7
Legends for movies S1 to S2

Other Supplementary Materials for this manuscript include the following:

Movies S1 to S2

1. Experimental Section

Materials and Devices: Chitosan (75%-85% deacetylation, medium molecular weight) and Gelatin (porcine skin-derived, Type A, molecular weight 50-100 kDa) were purchased from Sigma-Aldrich (USA). Concentrated hydrochloric acid and H_2O_2 were obtained from Shanghai Lingfeng Chemical Reagent Co., Ltd. Chitosan was purified to remove insoluble impurities and residual proteins before use. Gelatin did not require further purification before use. All cell-culture related reagents were purchased from Gibco (Grand Island, NY). Electro-deposition was performed in a CHI 660E three-electrode system, where the working electrode was a Ti plate (2 cm x 2 cm), the counter electrode was a Pt plate, and the reference electrode was Ag/AgCl.

For the preparation of FITC-labeled chitosan (FITC-Chit): Firstly, a 1% w/v chitosan solution is prepared by measuring 30 mL of the solution and pouring it into a culture dish with a diameter of 160 mm. It is then dried overnight in an oven to obtain a chitosan film produced by solvent evaporation. The film is then immersed in 30 mL of 1M NaOH for neutralization for 3 to 4 hours, followed by extensive washing with ultrapure water.

Under completely light-protected conditions, 2.5 mg of FITC is dissolved in 200 μL of DMF, followed by the addition of 800 μL of ethanol to obtain a FITC solution. A chitosan film from the aforementioned preparation is placed in each culture dish, to which 35 mL of 0.1M PB buffer (pH=7.4) and 30 μL of FITC solution are added. The reaction proceeds for 30 minutes. The labeled chitosan film (yellow-green in color) is thoroughly washed with ultrapure water and dissolved in hydrochloric acid (adjusted to pH=3). The solution is then neutralized to pH 9 with NaOH, the precipitate is collected, and it undergoes light-protected dialysis and lyophilization, finally resulting in the FITC-Chit raw material.

For the preparation of rhodamine B labeled gelatin (RBITC-Gel): First, a 1% w/v gelatin solution is prepared and its pH is adjusted to neutral with NaOH. Following the proportion used for FITC-labeled Chitosan molecules, an appropriate amount of RBITC is added to the gelatin solution. The mixture is then stirred for 30 minutes under light-protected conditions to react. After the reaction is complete, the solution undergoes light-protected dialysis and lyophilization, finally yielding the raw material.

In Situ Visual Monitoring of Electrodeposition in Fluidic Channel: A titanium plate and a platinum plate with a thickness of 0.05 mm were respectively used as the working electrode and the counter electrode. They were fixed on a quartz glass plate to form microchannels with a distance of 1.5 mm. The working area of the two electrodes is 2 mm × 2 mm.[1] The electrode pairs at both ends were respectively connected to the electrochemical workstation (CHI 660e) by wires. After the fluidic channel was filled with the electrolyte, electrodeposition was performed under a current density of 6.67 mA/cm^2. The hydrogel growth kinetics were observed and recorded under the opening mode of an inverted microscope.

Electro-fabrication of Chitosan and Chitosan/Gelatin Films: Chitosan (1% w/v) was stirred and dissolved overnight in 2M hydrochloric acid. After centrifugation and purification, the pH of the final control solution was adjusted to 4.5. Gelatin (1% w/v) was dissolved by stirring in a constant temperature water bath at 50°C. After cooling, it was mixed with the chitosan solution (1% w/v) in different volume ratios. Then, 0.1M H_2O_2 was added and stirred evenly to obtain the electrolyte for use. Electrodeposition

of chitosan or the mixture of chitosan and gelatin was performed at specified constant current densities (ranging from 1.67 to 13.33 mA/cm^2) and specified times. After electrodeposition, the electrodes coated with chitosan or chitosan/gelatin film were cleaned three times with ultra-pure water. The deposited chitosan or chitosan/gelatin film was then peeled off from the cathode to form independent films, which were subsequently freeze-dried for further characterization.

For BSA loading-release, in vitro degradation, in vitro cell and in vivo animal experiments, samples require chemical crosslinking before testing. Specifically, films prepared under designated conditions are freeze-dried at -40°C for 24 hours and then crosslinked with 0.5% w/v glutaraldehyde for 30 minutes. Subsequently, the crosslinked wet films are freeze-dried again for subsequent testing.

Characterization of films: The viscoelastic behavior of the hydrogel film was measured using a rotating rheometer (MARS III, HAAKE). Specifically, the viscoelastic behaviors of samples (2 cm diameter and 1 mm thickness) were measured by a rheometer at 37 °C. During the measurement process, the amplitude sweep test was first performed to define the linear viscoelastic region (LVR) in which the storage modulus is independent of the strain amplitude. The amplitude in LVR was selected for oscillation frequency sweep tests within 0.1-10 Hz. The measured storage modulus (G') represents the elastic properties of sample at this shear frequency, and the loss modulus (G'') represents viscous properties.

All fluorescence observations were performed using a confocal laser microscopy CLSM (Nikon A1R). The chemical and crystal structures of the freeze-dried films were analyzed using ATR-FTIR (Nicolet 5700, Thermo) and X-ray diffractometry (Rigaku D/max2550 VB/PC, Bruker). The surface and section morphology of different samples in the dry state were observed using FE-SEM (S-4800, Hitachi) under the condition of 15 kV. The Image Pro 5.0 software (Media Cybernetic, USA) was used to statistically estimate the aperture distribution and the thickness of the porous and dense layers based on the surface and cross-section images of the thin films. A gravimetric method was used for film's total porosity estimation,[2] the apparent density and porosity of the films were calculated by using Equations (1) and (2), respectively:[3]

$$\text{Apparent density } (g/cm^3) = \text{Mass of Film (g)} / [\text{Film thickness (cm)} \times \text{Film area } (cm^2)] \quad (1)$$

$$\text{Porosity (\%)} = [1 - \text{Apparent density } (g/cm^3) / \text{Bulk density } (g/cm^3)] \times 100\,\% \quad (2)$$

An ethanol-wicking technique to measure film porosity from interconnected pores.[5, 6] The porosity of the interconnected pores was calculated as the interconnected void volume over the total volume.

Experimentally, to determine the total volume, freeze-dried films were soaked in ethanol for 1h and weighted. A Kimwipe was then used to wick away ethanol within interconnected pores, and the films were weighted once again. The interconnected void volume was calculated as the volume of ethanol wicked from the films.

The mechanical properties of films (3.5 mm×20 mm strip samples, approximately 600 μm thick in dry state) were measured in both dry and hydrated states (after soaking in PBS for 2 hours to achieve water equilibrium) using a universal tensile test (CMT6104, 50 N sensor), at a stretching rate of $5mm*min^{-1}$.

The hydrophilicity of the films was analyzed using a contact angle tester (JC2000D2). Experimentally, water droplets (20 μL, containing 1mM Rhodamine) were placed on the surface of the dry film in air, and then the wetting behavior of the liquid on the film surface was observed.

The degradation test of the films involved cutting them into circular samples with a diameter of 10 mm. The samples were weighed and record the initial weight (M_0), then soaked in 5 ml of PBS containing Egg Hen lysozyme (5000 U/ml) at 37°C. At predetermined time points, the samples were removed from the liquid, rinsed, dried and weighed again to record the remaining weight (M_t). The weights of the samples were plotted against time to obtain the degradation profile of the films.

$$\text{Degradation ratio (\%)} = \frac{[\text{Initial weight } M_0(g) - \text{Remaining weight } M_t\ (g)}{\text{Initial weight } M_0\ (g)} \times 100\ \%$$

BSA loading and directional transport of Janus films: BSA containing PBS solution (pH about 7.4, 100 mg/mL) was firstly prepared, and the surface of the film was coated with PBS solution containing BSA (50 $\mu L/cm^2$) and then freeze-dried (note: the loading capacity was 5 mg/cm^2). The dried films were placed in the device shown in Figure 5 for BSA release testing. Sample collection was performed at different time points from the solution in both chambers, and the protein concentration was quantified using the BCA protein assay. Additionally, equal amounts of Coomassie Brilliant Blue G250 were added to both chambers for qualitative observation.

In Vitro Cell Studies: For the cell barrier estimation, the films were cut into circles with a diameter of 20 mm. They were then sterilized, fixed on a CellCrown™ (Sigma), and placed into a 24-well plate without touching the bottom of the well. L929 cells were re-suspended in culture medium at a density of 4.0×10^4 cells/mL. Then, 1 mL of cell-free culture medium was added to the well from outside the CellCrown™, and 900 μL of culture medium with 100 μL of cell resuspension was added onto the sample.

After incubating for 1, 4 and 7 days, live/dead staining (DOJINDO) was performed on the cells. The distribution of cells within the films was observed using confocal laser microscopy (CLSM, Nikon A1R). For the cell viability and proliferation tests, films were cut into circles with 10 mm in diameter, and then put into a 48-well plate. MC3T3-E1 were seeded onto the films at 5 × 10^4 cells per well and culture. The cell morphology was observed by SEM, and the proliferation rates of cells were evaluated using CCK8 assays (DOJINDO) at 1, 3 and 5 day post culture. For the cell osteogenic differentiation tests, cell seeding was the same as in the cell proliferation assay. After 7, 14, and 21 days of osteogenic incubation, the culture media were removed and the cells were washed with PBS. Then, 250 μL of 1% Nonidet P-40 (Beyotime) was added into each well and incubated at 37 °C for 1 h. 50 μL of the obtained cell lysate was added into 96-well plates, and then 50 μL of 1 mg/mL P-nitrophenylphosphate (Sangon) substrate solution was added. The mixed solution was incubated for 60 min. The ALP activity value was quantified at a wavelength of 405 nm using a microplate reader. ALP activity was expressed as 405 nm OD value/mg of total protein. The total protein concentration was determined using the BCA Assay Kits (Beyotime). ALP staining was performed using a BCIP/NBT ALP kit (Beyotime). Briefly, MC3T3-E1 cells were plated on the films in 48-well plates and cultured for 14 days. After fixation with 2.5% glutaraldehyde, the cells were incubated in a mixture of nitro-blue tetrazolium and 5-bromo-4-chloro-3-indolylphosphate. For the alizarin red S staining, after 21 days of osteogenic inducing culture, the MC3T3-E1 cells were fixed in 95% ethanol for 20 minutes and then incubated with 40 mM alizarin red S (ARS) staining solution (Sigma-Aldrich) for 20 minutes at room temperature. The unbound dye was removed with water washes.

In Vivo Studies: All procedures were approved by the Animal Research Committee (ECUST-2023-041). For subcutaneous implantation, to assess tissue compatibility of films and observe how the surrounding cells and tissue infiltrate into these films in vivo, a total of 12 SD rats (male, body weight 180 ± 5 g) were used. Films with a circular shape (a diameter of 7 mm) were sterilized using ethylene oxide gas (note: all films crosslinked by 0.5% w/v glutaraldehyde for 30 min in all in vivo experiments). The SD rats were randomly divided into four groups for different films' implantation. In order to alleviate the pain and panic of animals, anesthesia was carried out using sevoflurane by inhalation. The dorsal skin was shaved and sterilized with iodophor, and a 1.5 cm long incision was made using a blade mounted on a scalpel, followed by creation of two subcutaneous pockets using scissors. different samples were

slightly inserted into the formed pockets, respectively, and the incision was sutured with 3-0 nonabsorbable nylon sutures. After 4 and 8 weeks of implantation, respectively, the animals were subjected to euthanasia, and the implanted films were retrieved for gross observation and histological analysis. The H&E-stained and IHC-stained tissue samples were observed under optical microscopy and inflammation reactions, including cell and tissue invasion, were analyzed.

To perform a critical-sized calvarial defect model, a total of 15 8-week-old male SD rats were anesthetized by intraperitoneal injection of pentobarbital, then a 1.0~1.5 cm sagittal incision was made on the scalp, and the calvarium was exposed by blunt dissection. Two critical-sized defects were created per rat by using a 5 mm diameter trephine bur. Totally 30 defects are divided into 5 groups: no-film treated (n = 6), coverd with Dense Chit/Gelatin (n = 6), Janus Chit/Gelatin (n = 6) or the two different films loaded with 5 μg BMP-2. Then the wounds were carefully sutured. 4 and 6 weeks after operation, the rats were intraperitoneally injected with alizarin red (30 mg/ kg) and calcein (20 mg/ kg), respectively. After 8 weeks surgery, animals were sacrificed. Micro-CT, trichromatic sequential fluorescent labelingmethod and Van Gieson's staining were performed to observe the new bone regeneration. The specimens were scanned using a micro-CT system (mCT-80, ScancoMedical, Bassersdolf). The slice thickness was 20 mm and the threshold value was 225. Then, a 3D histomorphometric analysis was produced using the auxiliary histomorphometric software (Scanco Medical AG, Switzerland). The parameters of bone volume fraction (BV/TV), number of trabeculae (Tb.N), and bone mineral density (BMD) were compared in this study. For histological observation, the skulls were dehydrated in alcohols and embedded in PMMA. The sagittal section of the central area of each defect was cut and polished to a thickness of ~ 40 μm. The fluorescent labeling of the sections were observed using λex/λem of 543/580-670 nm (alizarin red, red) and 488/500-550 nm (calcein, green) on a Leica TCS SP8 microscope. Next, the sections were stained with Van Gieson's picrofuchsin for observation of new bone formation. The measurement of areas of newly formed bone was quantified using an image analysis system (Image Pro 5.0, Media Cybernetic, Silver Springs, MD)

Statistical Analysis: All data are presented as mean ± standard deviation (SD) of triplicates unless otherwise indicated. Statistical analyses were performed Origin (version 8.0) software using a two-tailed Student's t-test for two-sample comparison or one-way ANOVA with Tukey's multiple comparisons post hoc test. In these analyses, significant differences were accepted at $*p < 0.05$, $**p < 0.01$, $***p < 0.001$.

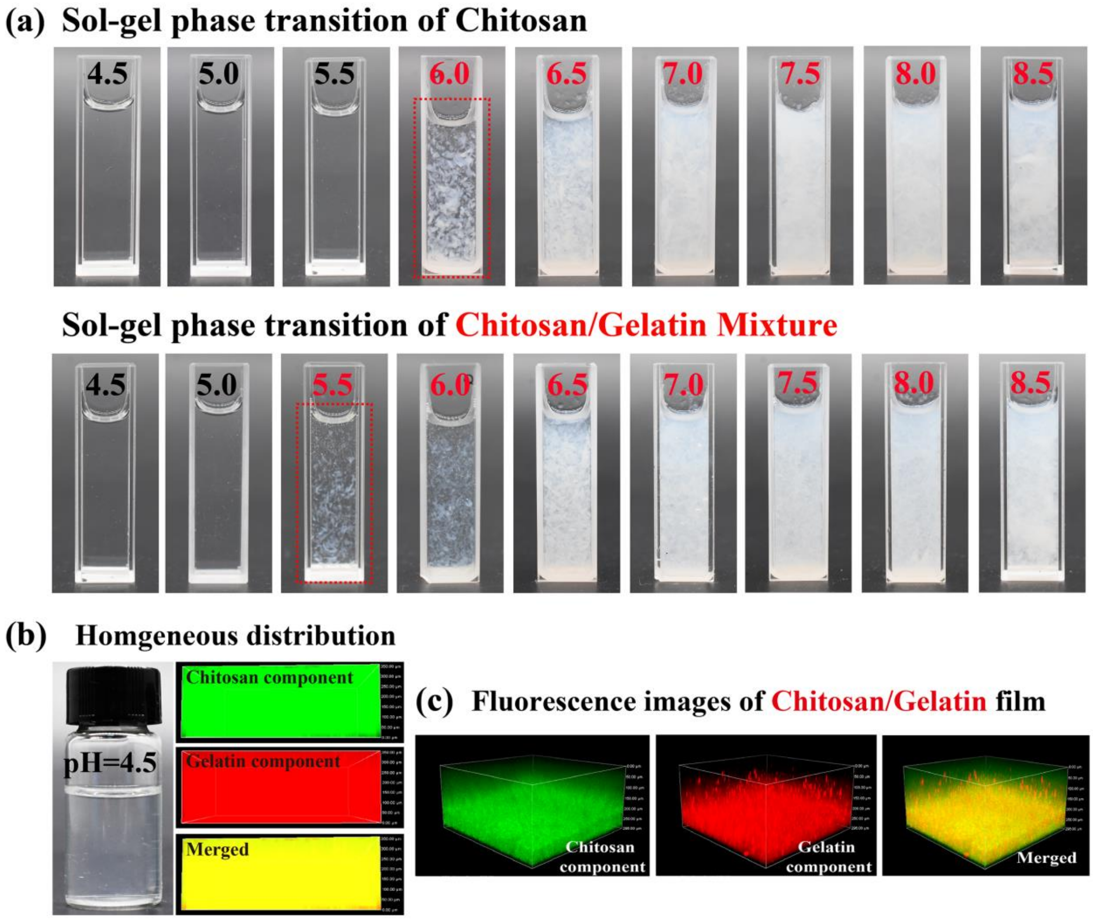

Fig. S1. (a) Images of 1% w/v chitosan solution and the mixture of chitosan and gelatin solution (both at 0.5% w/v) as a function of pH show that some aggregates appear in the gelatin-chitosan solution at pH 5.5. In contrast, chitosan does not precipitate until it reaches a higher pH (close to its pKa). (b) The picture of the chitosan and gelatin complex solution (0.5% w/v chitosan and 0.5% w/v gelatin, pH approx. 4.5) shows a transparent appearance, and the fluorescent confocal images further confirm the homogeneous distribution of chitosan (labeled with green fluorescence) and gelatin (labeled with red fluorescence) components in the solution. (c)The low magnification fluorescence images of the whole electro-assembled chit/gelatin film.

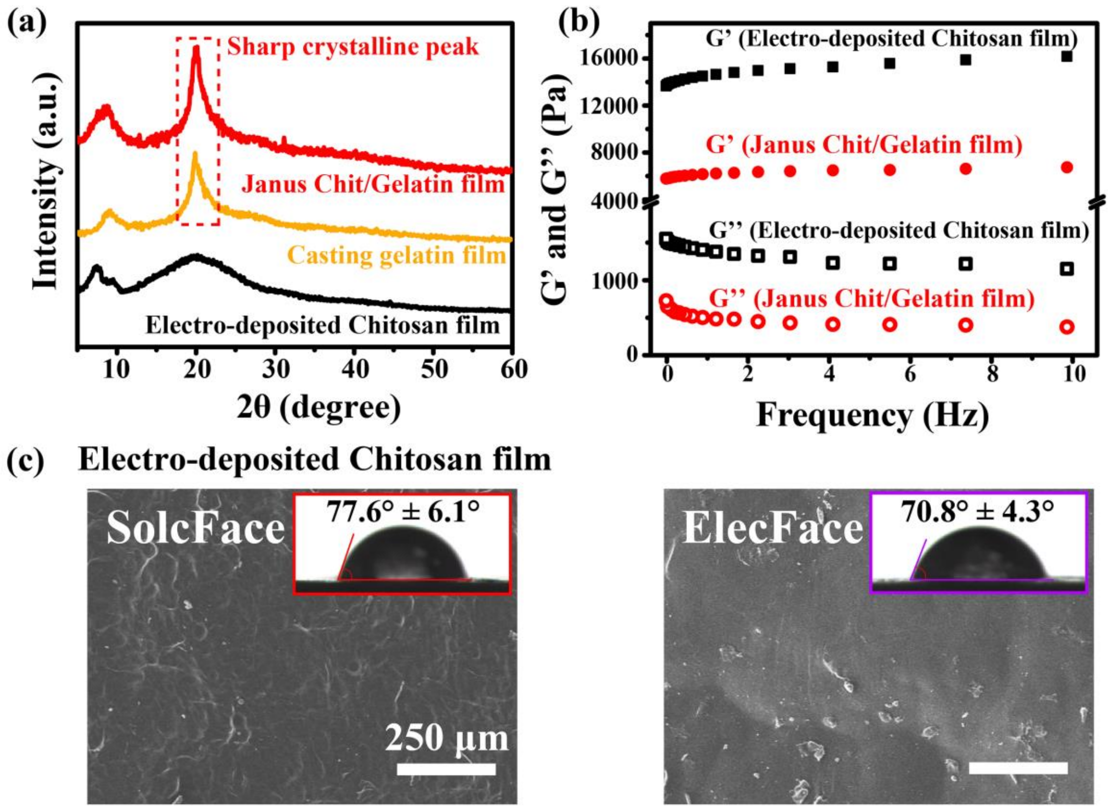

Fig. S2. (a) XRD of Janus Chit/Gelatin, chitosan film and casting gelatin filmsreveals that the Janus Chit/Gelatin film exhibits a sharp crystalline peak similar to the casting gelatin film, suggesting the presence of crystalline gelatin chains. (b) Rheological measurements indicate that the Janus Chit/Gelatin film has a decreased modulus compared to the chitosan film. (c) SEM images of the electro-deposited chitosan film indicate that the film has a non-porous dense structure, and both the dense ElecFace and SolcFace of electrodeposited chitosan control film showed a stable contact angle of about 70°.

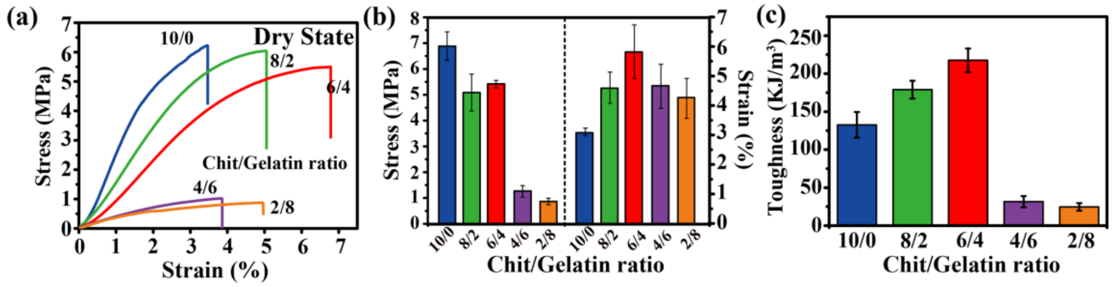

Fig. S3. The stress-strain curves of the Janus Chit/Gelatin films in the dry state, along with their quantitative measurements of ultimate stress, strain, and toughness, demonstrate the superior flexibility of the Janus Chit/Gelatin film when the Chit/Gelatin ratio is 6/4. (n=4) (Note: None of the samples described above have undergone chemical cross-linking).

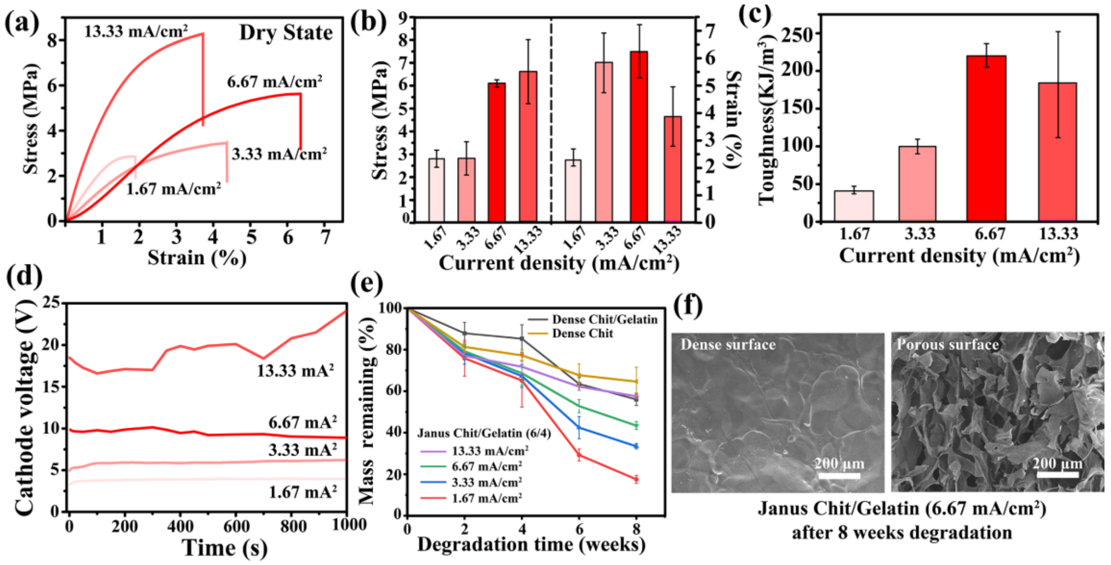

Fig. S4. (a-c) The dry stress-strain curves of uncrosslinked Janus Chit/Gelatin films, fabricated using different current densities, along with their quantitative measurements of ultimate stress, strain, and toughness, further demonstrate that the highest toughness of the Janus Chit/Gelatin film is achieved when the current density is 6.67 mA/cm^2. (n=4). (d) The electrostatic field strengthens or intensifies with increasing current density. (e) The degradation of crosslinked Janus Chit/Gelatin films was conducted in PBS solution containing Hen Egg lysozyme (5000 U/ml) at 37℃, n=6. (f) Morphology comparison of Janus Chit/Gelatin film (6.67 mA/cm^2) after 4 weeks degradation, indicating the dense layer remains non-porous structure.

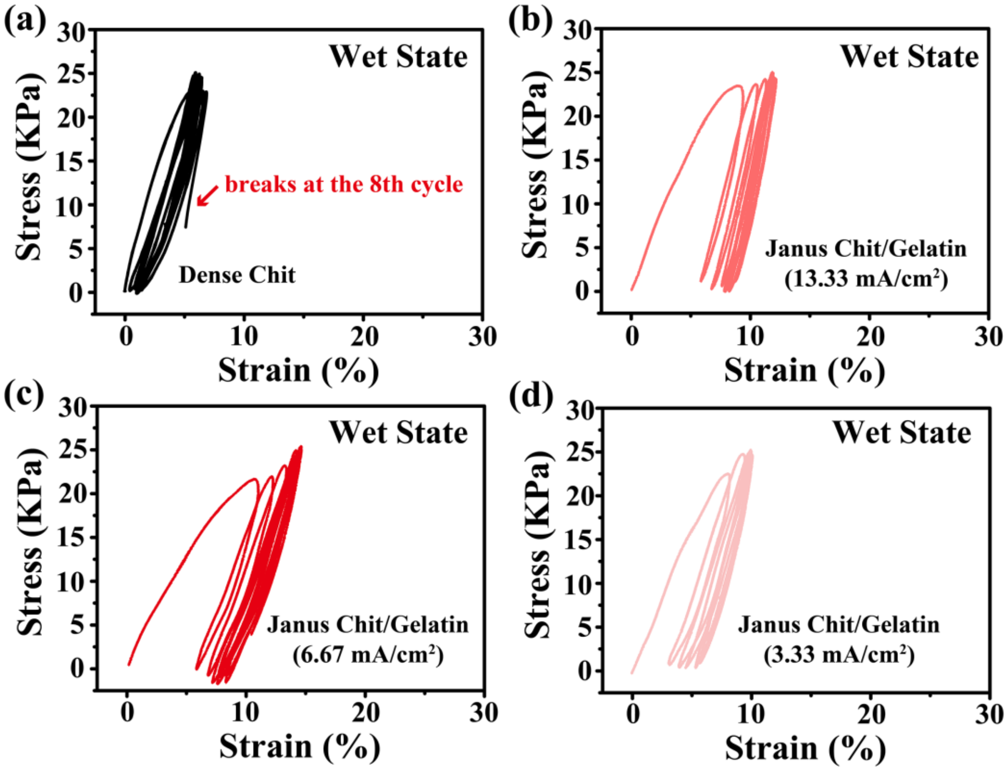

Fig. S5. The representative cyclic loading-unloading curves of Dense Chit film and Janus Chit/Gelatin films fabricated using different current density, which indicating the Dense Chit film exhibits brittleness, and breaks at the 8th cycle of loading-unloading. While the Chit/Gelatin Janus

films fabricated by different current density have stable mechanical properties, no fracture, mechanical degradation and significantly irreversible deformation were observed during cyclic loading. (Note: None of the samples described above have undergone chemical cross-linking).

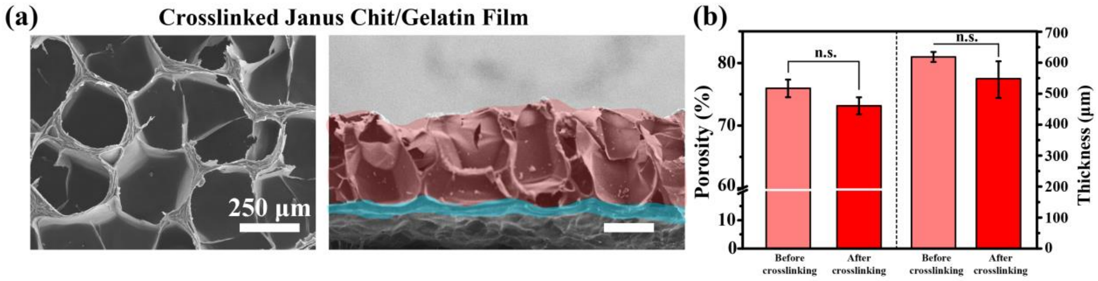

Fig. S6. (a) The surface and cross-sectional morphology of Janus Chit/Gelatin films (with a Chit/Gelatin ratio of 6/4, prepared at 6.67 mA/cm² for 1000 seconds) after crosslinking with 0.5% w/v glutaraldehyde for 30 minutes. (b) A quantitative comparison of the porosity and film thickness of Janus Chit/Gelatin films before and after crosslinking. (n=6).

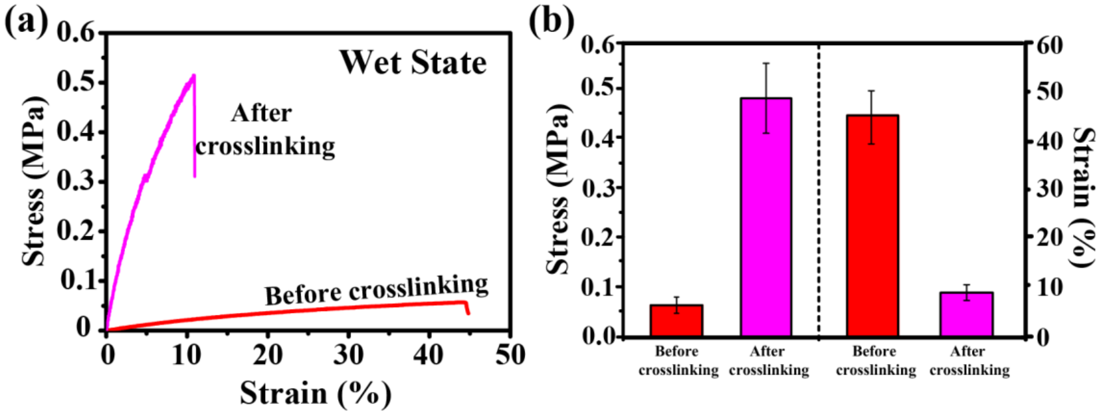

Fig. S7. The representative stress-strain curves of Chit/Gelatin Janus films (6/4) fabricated under a current density of 6.67 mA/cm^2 for 1000s before and after 0.5% glutaraldehyde crosslinking for 30 minutes. (n=4).

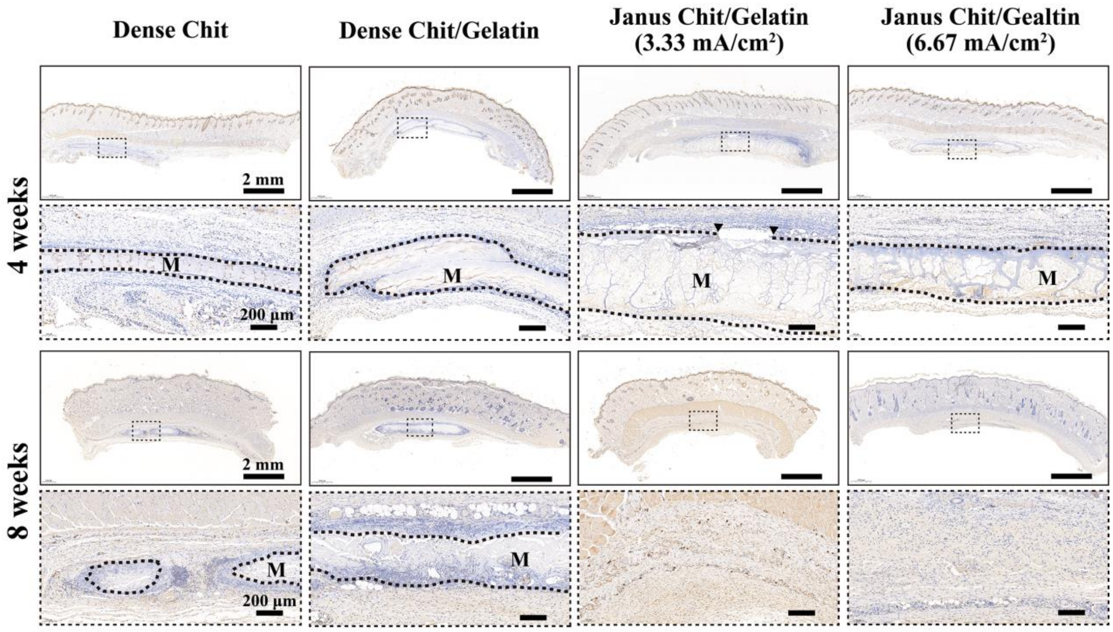

Fig. S8. Immunohistochemistry (IHC) staining of TNF-α with different magnifications of different films after subcutaneous implantation for 4 and 8 weeks, respectively (the black dotted line marks the implanted materials, M; the black triangle marks the dense layer of Janus porous film).(Note: All the films mentioned above were chemically cross-linked using 0.5%w/v glutaraldehyde for 30 min).

2. Supplementary Movies

Movie S1. Electrodeposition of chitosan/gelatin mixture solution

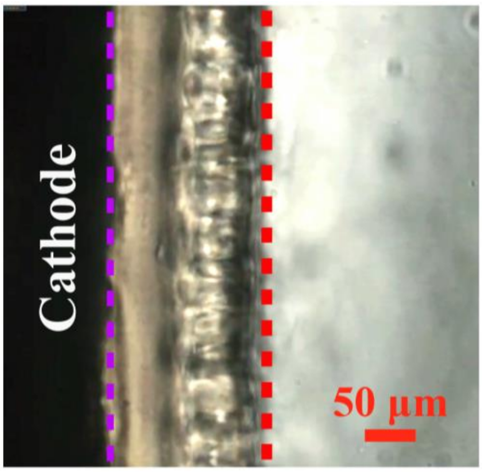

Movie S2. Electrodeposition of chitosan solution

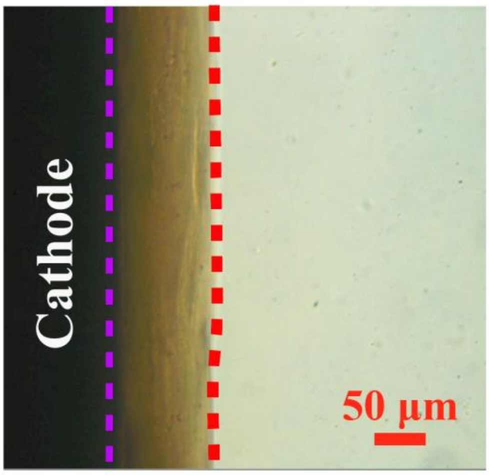

Reference

[1] Liu, Y.; Zhang, B.; Gray, K. M.; Cheng, Y.; Kim, E.; Rubloff, G. W.; Bentley, W. E.; Wang, Q.; Payne, G. F., *Soft Matter* **2013,** *9* (9), 2703-2710.

[2] Loh, Q. L.; Choong, C., *Tissue Engineering Part B-Reviews* **2013,** *19* (6), 485-502.

[3] Xue, J. J.; He, M.; Liu, H.; Niu, Y. Z.; Crawford, A.; Coates, P. D.; Chen, D. F.; Shi, R.; Zhang, L. Q., *Biomaterials* **2014,** *35* (34), 9395-9405.

[4] Ruan, J.; Wang, X.; Yu, Z.; Wang, Z.; Xie, Q.; Zhang, D.; Huang, Y.; Zhou, H.; Bi, X.; Xiao, C.; Gu, P.; Fan, X., *Adv. Funct. Mater.* **2016,** *26* (7), 1085-1097.

[5] Macaya, D.; Spector, M., *Biomedical Materials* **2012,** *7* (1).

[6] Luo, R. C.; Wu, J.; Dinh, N. D.; Chen, C. H., *Advanced Functional Materials* **2015,** *25* (47), 7272-7279.
